# Novel syntrophic bacteria in full-scale anaerobic digesters revealed by genome-centric metatranscriptomics

**DOI:** 10.1038/s41396-019-0571-0

**Published:** 2020-01-02

**Authors:** Liping Hao, Thomas Yssing Michaelsen, Caitlin Margaret Singleton, Giulia Dottorini, Rasmus Hansen Kirkegaard, Mads Albertsen, Per Halkjær Nielsen, Morten Simonsen Dueholm

**Affiliations:** 1 Center for Microbial Communities, Department of Chemistry and Bioscience, Aalborg University, Aalborg, Denmark; 2 State Key Laboratory of Pollution Control and Resource Reuse, College of Environmental Science and Engineering, Tongji University, Shanghai, PR China

**Keywords:** Bacterial genetics, Transcriptomics, Next-generation sequencing, Microbial ecology, Metagenomics

## Abstract

Short-chain fatty acid (SCFA) degradation is an important process in methanogenic ecosystems, and is usually catalyzed by SCFA-oxidizing bacteria in syntrophy with methanogens. Current knowledge of this functional guild is mainly based on isolates or enrichment cultures, but these may not reflect the true diversity and in situ activities of the syntrophs predominating in full-scale systems. Here we obtained 182 medium to high quality metagenome-assembled genomes (MAGs) from the microbiome of two full-scale anaerobic digesters. The transcriptomic response of individual MAG was studied after stimulation with low concentrations of acetate, propionate, or butyrate, separately. The most pronounced response to butyrate was observed for two MAGs of the recently described genus *Candidatus* Phosphitivorax (phylum Desulfobacterota), expressing a butyrate beta-oxidation pathway. For propionate, the largest response was observed for an MAG of a novel genus in the family Pelotomaculaceae, transcribing a methylmalonyl-CoA pathway. All three species were common in anaerobic digesters at Danish wastewater treatment plants as shown by amplicon analysis, and this is the first time their syntrophic features involved in SCFA oxidation were revealed with transcriptomic evidence. Further, they also possessed unique genomic features undescribed in well-characterized syntrophs, including the metabolic pathways for phosphite oxidation, nitrite and sulfate reduction.

## Introduction

Microbial syntrophy describes the obligatory mutualistic metabolism that occurs between two or more microorganisms, where the metabolic end-product of the primary metabolizer is immediately consumed as substrate by the others. This shifts the equilibrium of the catabolic reaction in the primary metabolizer which would otherwise not yield enough energy to support growth [1–4]. Syntrophic fatty acid oxidizing bacteria play an essential role in the conversion of short-chain fatty acids (SCFAs) such as butyrate and propionate to methanogenic precursors (acetate, H_2_, and formate). This conversion accounts for much of the carbon flux in methanogenic ecosystems [5], for instance, the full-scale anaerobic digesters (ADs) treating biological sludge with recovery of biogas that are widely used in wastewater treatment plants (WWTPs). The SCFA metabolism can only occur when methanogens rapidly consume the produced methanogenic precursors [3, 6]. Due to the fastidious metabolism type, these syntrophic bacteria can only grow at low rates and yield [7] and are commonly found in low abundance [8, 9]. Therefore, syntrophic SCFA degradation can easily become a bottleneck step for the anaerobic digestion process. Disturbances at this step would induce SCFA accumulation and performance instability, which frequently occur during operation of full-scale ADs [10, 11]. Hence, a better understanding of this functional guild might ultimately improve the digestion efficiency.

Our current knowledge of syntrophic bacteria involved in SCFA oxidation is mostly based on studies performed on a few model organisms such as *Syntrophomonas wolfei*, *Syntrophobacter fumaroxidans*, and *Pelotomaculum thermopropionicum* incubated as mono- or defined co-cultures [2, 12] or in enrichments [13]. However, these well-characterized syntrophic butyrate or propionate oxidizers were not detected as core populations in previous surveys of full-scale ADs [8, 9, 14–16], and other taxa may participate in SCFA degradation as indicated by stable isotope probing of nucleic acids or enrichment experiments [13, 17, 18]. It is therefore important to identify the key bacterial members directly and actively involved in SCFA oxidation in full-scale systems.

The functional importance of specific microbial taxa in complex communities can be characterized using a genome-guided metatranscriptomic approach. This has previously been applied to identify taxa involved in the anaerobic degradation of cellulosic biomass [19] and oleate [20] using lab-scale enrichments. The transcriptomic response of individual taxon can also be measured in response to a defined environmental stimulus [21]. When combined with short-term incubations, this may provide a direct link between the native members of microbial communities and the stimulus-related function.

To identify the key SCFA degrading bacteria and elucidate their activities in full-scale systems, we obtained metagenome-assembled genomes (MAGs) from two different full-scale ADs in Denmark that represent typical anaerobic digestion systems treating waste activated sludge at WWTPs. We then applied a genome-centric metatranscriptomic approach to study the change of transcriptomes of targeted microbial members in response to SCFA stimuli. A comprehensive dataset from this study enabled the discovery of novel syntrophic bacterial species, and manually curated genome annotation revealed the energy conservation metabolisms related to SCFA oxidation in the three most responsive members.

## Materials and methods

### SCFA stimuli experiments

The two full-scale ADs investigated (Table S1) are located at two municipal WWTPs (at Randers and Fredericia, Denmark) and have been stably operated at mesophilic condition for more than 5 years. The digester at Randers treats surplus activated sludge and primary sludge, and the digester at Fredericia uses surplus activated sludge pretreated by thermal hydrolysis process (THP) as feedstock. Slurry directly taken from these digesters was immediately used for the lab-scale SCFA stimulation experiments, which were performed in the automatic methane potential test system (AMPTS) II (Bioprocess control, Lund, Sweden) at temperature and stirring conditions identical to those in full-scale digesters. Each 500 mL reactor was filled with 400 mL freshly collected digester slurry at the volatile solids concentrations of 22.8 g/L (Randers) and 25.2 g/L (Fredericia), flushed with N_2_ gas for 20 min and immersed in a water bath (38 ± 0.5 °C). The slurry from each digester was incubated for about 3 days anaerobically with continuous stirring. Each incubation included an initial starvation period of 17 h to exhaust the residual carbon in the fluid. To stimulate the microbial community, 4 mL of a concentrated SCFA solution (acetate, propionate, or butyrate separately) was added into individual reactors after the starvation period to reach a final concentration of 3 mM for acetate, and 2 mM for propionate and butyrate. The same volume of distilled water was used as a negative control. Experiments were performed in triplicate reactors for each SCFA and in duplicate for the control. During the SCFA degradation periods, sludge fluid was sequentially sampled and immediately frozen in liquid nitrogen, and preserved at −80 °C in several aliquots for further use. SCFA concentration and methane yield during incubation were analyzed as detailed in Supplementary Methods. The SCFA stimulation was repeated 24 h after starting the first stimuli event to confirm the reproducibility of the SCFA degradation behavior.

### Metagenomic analyses

Slurry samples originating from digesters at Fredericia and Randers WWTPs were used for DNA extraction and metagenomic sequencing (Data S1, Supplementary Methods Section 1.2). The raw metagenomic sequences were trimmed and assembled with CLC Genomics Workbench v.9.5.2 (QIAGEN Bioinformatics), generating 12 single-assemblies and 6 co-assemblies (Table S2). Details are provided in Supplementary Methods.

The assemblies and associated mapping data were exported as FASTA and BAM files, respectively. Script jgi_summarize_bam_contig_depths from the MetaBAT2 package was used to calculate coverage from the BAM files for each assembly. Metagenomic binning was applied to both single- and co-assemblies using MetaBAT2 v.2.12.1 [22], with options –minContigLength 2000, –minContigDepth 2. This resulted in 491 bins for the digester at Fredericia WWTP and 1007 for that at Randers WWTP. The bins from each digester were dereplicated to produce medium to high quality MAGs using dRep v.2.2.1 [23] with options dereplicate_wf -p 16 -comp 50 -con 25.

The completeness and contamination of each MAG were assessed based on the presence of lineage-specific, conserved, single-copy marker genes using CheckM v.1.0.11 [24]. Objective taxonomic classifications were assigned to the MAGs according to the Genome Taxonomy DataBase (GTDB) taxonomy (release 03-RS86) using the toolkit GTDB-Tk (v.0.1.3) with the classify workflow [25]. A phylogenetic genome tree of the bacterial MAGs was created and refined as detailed in Supplementary Methods.

The MAGs were annotated using Prokka (v.1.12) [26] with the bacteria or archaea database based on their taxonomic classification [25]. An *e*-value threshold of 10^−6^ was used for prediction of coding sequences (CDSs). The CDSs and contigs of each MAG were labeled with the MAG name and merged to create the CDS and MAG reference database for further metatranscriptomic analysis. The entire CDS set of the MAGs was further analyzed using EnrichM (v.0.2.0) (https://github.com/geronimp/enrichM), and annotated with Kyoto Encyclopedia of Genes and Genome (KEGG) orthologous group ids (KO) for ensuing metabolic pathway analysis [27]. KOs involved in oxidation of propionate via the methylmalonyl-CoA pathway, butyrate via the beta-oxidation pathway, and acetate via the reversed Wood–Ljungdahl pathway [28], were selected based on a list of gene families (determined by KO) which are directly associated with the corresponding KEGG metabolisms (Fig. S1, Data S2).

In order to estimate the relative abundance of each MAG, we calculated the fraction of trimmed metagenomic reads which were mapped (>95% sequence identity, 100% read-length) to the contigs of each MAG (Table S3, Data S3).

### Genome-centric metatranscriptomic analyses

Total RNA was extracted from the samples of two time points, i.e., 1 h before and 1 h after the first SCFA addition in three biological replicates, and rRNA was depleted before metatranscriptomic sequencing. Finally, 30 metatranscriptomes were generated (Data S1, Supplementary Methods Section 1.3). The cDNA reads, after trimming of raw reads and further removal of detected rRNA, were considered as the mRNA sequences for later use.

The processed mRNA reads were mapped to the CDS reference database made from the constructed MAGs using the CLC Genomics workbench (identity of ≥98% and ≥80% of the read length). Datasets originating from Fredericia or Randers were studied separately (Table S4, Data S3). The transcriptomic data (reads mapped per CDS) were analyzed in R v.3.5.0 [29] using the DESeq2 v.1.20.0 differential gene expression workflow [30]. The fold change (FC) and *p* value were calculated for the expression of individual CDS after each SCFA stimulation relative to the baseline before stimuli. CDSs with FC ≥ 2 and *p* value < 0.05 or FC ≤ 0.5 and *p* value < 0.05 were described as “upregulated” or “downregulated” genes. Prior to analysis, CDSs with less than five reads mapped were removed due to a low signal-to-noise ratio. 61 gene families determined by KO, which were related to oxidation of propionate, butyrate, and acetate (Data S2), were selected as targets to further analyze the different SCFA-oxidation metabolisms in the microbiome. The plots and heatmaps were made using the ggplot2 package v.3.1.0 [31].

### Amplicon sequencing analysis

Bacterial community composition was investigated based on previous data derived from amplicon sequencing the V1-3 variable regions of the bacterial 16S rRNA genes [8]. Raw sequences were processed with usearch v.10.0.240 [32] in order to generate amplicon sequence variants (ASVs) and an ASV table. To evaluate the relative read abundance of the three novel syntrophs described in this study, nearly full-length 16S rRNA gene sequences associated with the MAGs were extracted and used as reference. ASVs were mapped to the full-length 16S rRNA sequences using usearch_global with a minimum identity of 94.5%, and annotated based on their percentage identity to the reference sequences with the thresholds for genera (94.5%) and species (98.7%) proposed by Yarza et al. [33]. Amplicon data were further analyzed using Ampvis2 [34]. Both novel and previously described syntrophic bacterial genera [3, 35] were specifically analyzed. For details see Supplementary Methods.

### Genome curation and improved annotation

Three bacterial MAGs demonstrating strong positive responses to SCFA stimuli were selected for further genome curation and annotation. Genome annotation was performed in the ‘MicroScope’ annotation pipeline (v.3.12.0) [36]. Automatic annotations were validated and curated manually for the genes involved in metabolic pathways of interest with the assistance of the integrated MicroCyc [37] and KEGG [27] databases. The hydrogenases identified were further checked and classified using the HydDB tool [38].

## Results and discussion

### Stimulation of the microbiome by SCFAs

**Fig. 1:**
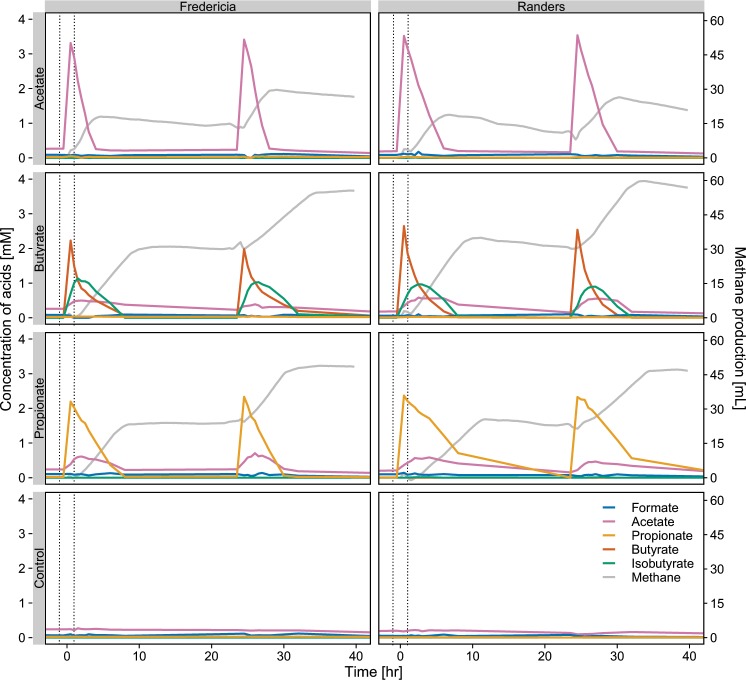
Concentration of acids and net production of methane before and after the SCFA stimuli events. Sludge slurries originating from digesters at Fredericia and Randers were used. The vertical dotted lines indicate the time points where samples were retrieved for transcriptomic analysis. Distilled water was added instead of SCFA solution for the control. Net methane production from the added SCFAs was calculated by subtracting the average total methane yield of the control reactors from that of the reactors stimulated with SCFAs, and the volume was corrected to standard conditions (20 °C, 1 atm).

Biomass from full-scale mesophilic digesters at Fredericia and Randers WWTPs were incubated in lab-scale reactors to investigate the response of the microbial communities to the addition of acetate, propionate, and butyrate separately, after a preincubation without feeding to exhaust the residual carbon. The conversion of all SCFAs started immediately after addition and showed the same conversion patterns for the microbiome from both digesters (Fig. 1). Acetate and propionate were consumed following zero-order kinetics, whereas butyrate followed first-order kinetics. Methane production occured with the degradation of SCFAs. Generation of acetate accompanied the degradation of propionate and butyrate. Furthermore, for butyrate, first accumulation and later consumption of isobutyrate occurred, indicating interconversion of the two isomers catalyzed by the active microbes in AD systems as previously observed [39, 40]. The degradation patterns were reproduced in each reactor following a second SCFA stimulus. This demonstrated high similarity between the two digesters in terms of microbial transformations, and is in accordance with previous results of similar SCFA-degradation experiments [10, 41, 42].

### One hundred and ninety-eight draft genomes derived from metagenomics

**Fig. 2:**
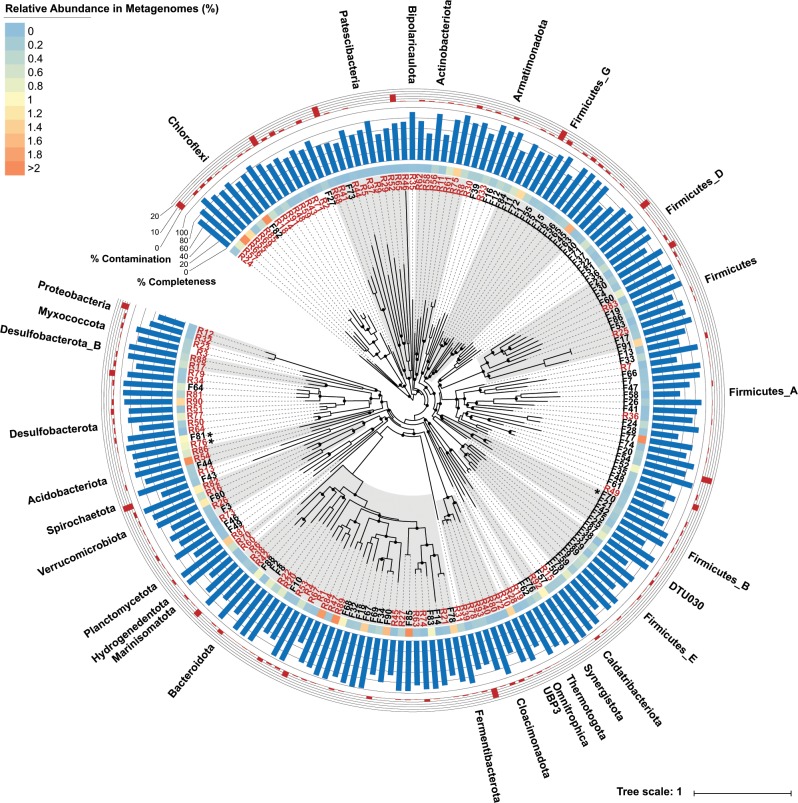
Phylogenetic genome tree of the bacterial MAGs reconstructed from two full-scale anaerobic digesters at Fredericia (black) and Randers (red). Bootstraps >70% are indicated by the solid black circles. Tree scale indicates evolutionary distance as rate of substitution per site. The MAGs F70, F81, and R76 are indicated by the stars. The average abundance of each MAG in the corresponding digester is shown in the inner circle heatmap. Completeness and contamination of each MAG determined by CheckM are shown in the blue and red barplots, respectively.

We sequenced 12 metagenomic libraries from biomass samples originating from the two full-scale digesters and the SCFA stimuli experiments (Data S1). This yielded 66.6 giga bp of paired-end Illumina data after quality filtering (Table S3). Metagenomic binning and further dereplication of the genome bins resulted in the recovery of 95 and 103 MAGs (≥50% completeness, ≤25% contamination) from the digesters at Frederica and Randers, respectively (Data S3). About 60% and 40% of the DNA reads could be mapped to the 95 Fredericia MAGs and 103 Randers MAGs, respectively (Table S3). We therefore assume that the obtained MAGs provide a fair representation of the complete microbial communities. The MAGs included 184 bacterial and 14 archaeal genomes spanning 30 bacterial and 4 archaeal phyla (Data S3). For the bacterial community, a large clade (57 out of 95) of Fredericia MAGs were assigned to the highly diverse Firmicutes phylum, while the Randers MAGs were more evenly distributed in different phyla including Bacteroidetes, Chloroflexi, Desulfobacterota, Firmicutes, and Patescibacteria (Fig. 2). This difference in bacterial composition was consistent with previous results from 16S rRNA gene amplicon analyses [8].

### Transcriptional responses of MAGs to SCFA stimuli

In order to identify MAGs responding to the SCFA stimuli, the transcriptomic responses were investigated using samples collected an hour before and an hour after the first SCFA addition for each microbiome. Thirty rRNA depleted metatranscriptomic libraries were sequenced from the two digesters under five different conditions before and after the stimuli events (Data S1). The libraries generated 11.8 million (Frederica) and 18.5 million (Randers) reads after quality filtering. mRNA reads were mapped to the predicted CDSs of the MAGs from the corresponding digester, resulting in 9.6–13.8% reads mapped to the Fredericia CDSs and 9.5–12.6% to the Randers CDSs (Table S4). Reduced mapping percentage of mRNA reads compared with metagenomic reads is common in genome-centric metatranscriptomics [19, 43]. This is partly a result of setting more stringent mapping criteria for mRNA, but the error prone automated identification of CDS may also play a role.

**Fig. 3:**
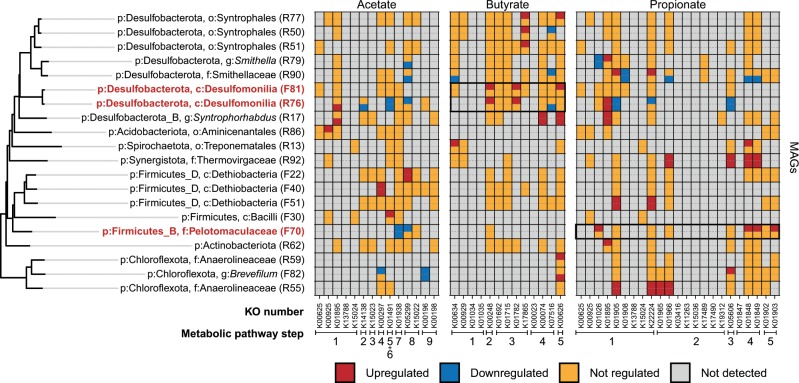
Transcriptional responses of MAGs after the SCFA stimulation. To the left is a phylogenetic tree of the bacterial MAGs which demonstrated upregulation of any of the genes directly involved in oxidation of acetate, butyrate, and propionate after stimulation by the corresponding SCFA. It is derived from the overall genome tree in Fig. 2. The heatmap to the right shows the transcriptional responses of genes involved in the bioconversion of SCFAs in the bacterial MAGs. 13, 19, and 31 gene families determined by KEGG ontology (KO) numbers were used, representing the key genes directly involved in the aforementioned pathways, which can catalyze bioconversion in 9, 5, and 5 steps (as defined in Fig. S1 and Data S2). The heatmap is colored according to fold change (FC) and the corresponding *p* value (not corrected for multiple testing to increase sensitivity) of CDSs for the given KO and are categorized as follows: Downregulated: FC ≤ 0.5 and *p* value < 0.05; Upregulated: FC ≥ 2 and *p* value < 0.05; Not regulated: 0.5 < FC < 2 or *p* > 0.05; Not detected: no expression or not encoded. For each MAG, the corresponding SCFA stimuli and control conditions are shown in the upper- and lower-half of the tile, respectively. The three MAGs in bold red were expressing ≥80% of the bioconversion steps in the associated pathways highlighted in black squares, with ≥3 steps upregulated (two steps for R76). The columns represent different genes (KOs), which are clustered into steps based on similar functions in the pathway.

Key genes directly involved in syntrophic oxidation of propionate, butyrate, and acetate (Data S2) were found to be expressed in 157 MAGs spanning 29 bacterial phyla (Fig. S2). However, increased transcription of these genes after stimulation by the corresponding SCFA was only observed in 20 MAGs (Fig. 3). Eight of the MAGs belonged to the Desulfobacterota and Desulfobacterota_B phyla, which include the bacterial genera *Smithella* and *Syntrophorhabdus* that comprise typical known syntrophic bacteria.

We identified two bacterial MAGs that displayed strong positive responses to SCFAs, which expressed genes for more than 80% of the bioconversion steps (Data S2) in the SCFA oxidation pathways and significantly upregulated genes in at least three of the steps after SCFA addition. They were MAG F70 for propionate, and MAG F81 for butyrate. In addition, we included R76 in the following analysis as it is phylogenetically closely related to F81 and significantly upregulated two successive steps after butyrate addition. No bacterial MAGs responded to acetate as strongly as to propionate and butyrate. Other bacterial MAGs with low but significant positive responses to SCFA addition and the responses of archaeal members are described in the Supplementary Notes.

To gain a comprehensive understanding of the physiology and ecology of the three putative SCFA oxidizers discovered here, manually curated genome annotation and metabolic pathway reconstruction were performed, and expression of related genes was further analyzed.

### Butyrate oxidation via two *Candidatus* Phosphitivorax species (F81 and R76)

Genome-based taxonomy analysis assigned MAGs F81 and R76 to the same genus as the recently discovered *Ca*. Phosphitivorax anaerolimi strain Phox-21 (See Supplementary Notes), which was not previously described as a syntrophic butyrate oxidizing bacterium (SBOB) [44]. Based on the following genomic and transcriptomic characteristics, we propose *Ca*. Phosphitivorax anaerolimi F81 and *Ca*. Phosphitivorax butyraticus R76 for naming the two MAGs.

#### Butyrate beta-oxidation

**Fig. 4:**
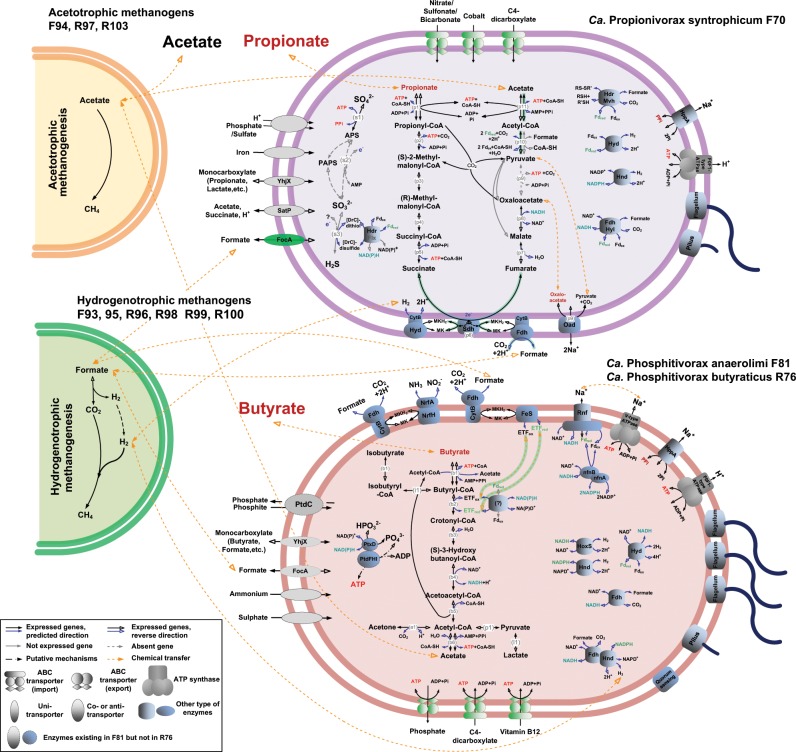
Metabolic pathways in *Ca*. Propionivorax syntrophicum F70, *Ca*. Phosphitivorax anaerolimi F81 and *Ca*. Phosphitivorax butyraticus R76, and their interactions with methanogens. The pathways are constructed based on the annotated genome sequences (Data S4). Orange and green cell cartoons symbolize MAGs of *Methanothrix* (F94, R97, R101, R103) consuming acetate and MAGs of *Methanoculleus* (F93, F95, R98, R99) and Methanoregulaceae (R100) utilizing H_2_ and formate produced by the bacteria. Only the SCFA oxidation pathways with the related electron-transfer systems, transporters, and the potential chemolithotrophic metabolisms are shown for the bacterial MAGs. A more detailed metabolic description is provided in Fig. S3. The reactions and uni-transporters highlighted in green indicate that expressions of the related genes were upregulated after addition of the corresponding SCFA, with fold change > 2 and *p* value < 0.05. Rnf: *Rhodobacter* nitrogen fixation complex; Nfn: NADH-dependent Fd_red_:NADP^+^ oxidoreductase.

A complete butyrate beta-oxidation pathway was reconstructed for MAGs F81 and R76 (Fig. 4). Therein, butyrate can be activated to butyryl-CoA by the ATP-consuming acyl-CoA synthetases, of which the expression of a specific butyrate-CoA ligase was detected. Acetyl-CoA transferases can sacrifice one acetyl-CoA to activate butyrate without energy consumption, but such genes were not highly expressed. The produced butyryl-CoA was further converted to crotonyl-CoA with acyl-CoA dehydrogenases, of which a specific butyryl-CoA dehydrogenase (Bcd) was upregulated (1.7-fold for F81 and 2.5-fold for R76) following butyrate addition. Further oxidation proceeds via 3-hydroxybutyryl-CoA to acetoacetyl-CoA with the bifunctional enoyl-CoA hydratase/3-hydroxyacyl-CoA dehydrogenases, that were elevated (up to 2.2-fold for F81 and 3.0-fold for R76) after butyrate addition. The acetoacetyl-CoA was cleaved to two acetyl-CoA moieties with the acetyl-CoA acetyltransferases (upregulated up to 2.3-fold for F81 and 3.5-fold for R76), which can be further transformed to acetate with ATP production via the acetyl-CoA synthetases. Both MAGs encoded multiple paralogs for each step, with only some of these being upregulated for most steps after butyrate addition, which was similar to the well-characterized SBOBs, such as *S. wolfei* [45] and *Syntrophus aciditrophicus* [46].

A comparative genomic analysis demonstrated that the beta-oxidation pathway also exists for *Ca*. Phosphitivorax anaerolimi strain Phox-21 (Data S5), but was not discovered by Figueroa et al. [44], perhaps due to the automatic annotation pipeline applied therein.

#### Electron flow from acyl-CoA dehydrogenases

Beta-oxidation of each molecule of butyrate results in net synthesis of one ATP. In addition, two sets of electrons are generated at two different redox potentials: the first pair is generated during the oxidation of butyryl-CoA to crotonyl-CoA in the form of reduced electron transfer flavoprotein (ETF) and the second pair is generated from the oxidation of 3-hydroxybutyryl-CoA to acetoacetyl-CoA (E^0′^ = −250 mV) which is directly used to reduce NAD^+^. The first electron pair have high redox potential (E^0′^ = −125 mV), which can be only used by the membrane complexes utilizing chemiosmotic energy (such as the Fe–S oxidoreductase, the Fix complexes) in *S. wolfei* and *S. aciditrophicus* [7, 45, 46]. Such complexes can transfer the electrons from the reduced ETF to menaquinone (MK) with inward translocation of protons, and the reduced form: menaquinol (MKH_2_) can be further oxidized by the membrane bound, externally oriented formate dehydrogenases or hydrogenases with translocation of two other protons.

F81 and R76 encoded and expressed three gene clusters related to ETF, but seem to lack the Fix complex. The Fe–S oxidoreductase-EtfAB complex, including ETF alpha-, beta-subunits, and a membrane-bound Fe–S oxidoreductase (lacking in R76 probably due to genome incompleteness), was clustered with the beta-oxidation specific genes and actively expressed with butyrate. The other two gene clusters related to ETF subunits both clustered with acyl-CoA dehydrogenases (*bcd/etfAB*), which could reduce NAD^+^ with electrons derived from butyryl-CoA and reduced ferredoxin (Fd_red_) [7], but demonstrated much fewer transcripts compared with the aforementioned one.

#### H_2_/formate production and other confurcation/bifurcation mechanisms

Three externally oriented formate dehydrogenases *fdoIGH-fdhA* [47, 48] were encoded, one of which was actively expressed. Each of these gene clusters encoded a membrane-bound b-type cytochrome, which transfers the electrons from MKH_2_ to an Fe–S protein, the catalytic subunit of the formate dehydrogenases, and generates formate in the periplasm, similar as that described for *S. wolfei* [7, 49]. Three cytoplasmic formate dehydrogenases (FDH4-6) and four putative hydrogenases (HYD1-4) were also detected. FDH5 contains subunits of NADH-quinone oxidoreductase, which was predicted to catalyze formate production from electron bifurcation between Fd_red_ and NADH [7, 45]. HYD2-4 (*hndABCD*) were proposed to catalyze the reversible H_2_-driven NADP^+^ reduction [50]. Among these hydrogenases, HYD4 was adjacent to FDH4, forming a large hydrogenase/formate dehydrogenase gene complex. This complex might catalyze the exchange between H_2_ and formate generated from NAD(P)H, similar to the complex found in *S. wolfei* [6]. Expression of another cytoplasmic NAD-reducing hydrogenase (HoxS gamma subunit) indicated active H_2_ formation from NADH. The produced H_2_ can spontaneously diffuse through the cell membrane, while formate in the cytoplasm can be exported by the formate transporters (FocA and an oxalate/formate antiporter). F81 and R76 also encoded Rnf (*rnfABCDEG*) and Nfn (*nfnAB*) complexes, which can catalyze electron confurcation/bifurcation between NADH, NADPH, and Fd_red_. Both complexes could thus be a source of Fd_red_ for formate production, but were not actively expressed.

#### Inactive phosphite oxidation and CO_2_ fixation metabolisms

*Ca*. Phosphitivorax anaerolimi strain Phox-21 was described to live a chemolithoautotrophic life by obtaining energy from phosphite oxidation and fixing CO_2_ via a proposed reductive glycine pathway [44]. In our study, the entire *ptx-ptd* gene cluster for dissimilatory phosphite oxidation was reconstructed for F81 (but not found in R76, which could be due to a low MAG completeness of 79.6%). Only the genes involved in the reductive glycine pathway were found for inorganic carbon assimilation in both MAGs (Fig. S3, Data S4), while essential genes for other autotrophic CO_2_ fixation pathways were lacking. However, these genes were not actively expressed under any condition, indicating that these microorganisms did not live as autotrophs, but probably as heterotrophic syntrophs, unlike *Ca*. Phosphitivorax anaerolimi strain Phox-21, which was enriched with externally added phosphate and CO_2_ [44].

#### PMF and ATP synthesis

F81 and R76 may generate proton motive force (PMF) with the membrane-embedded pyrophosphatase and Rnf complex, or could export protons via dissimilatory nitrite reduction coupled to formate oxidation as catalyzed by nitrite reductase and relevant formate dehydrogenase. Nevertheless, only the pyrophosphatase was significantly upregulated (2.3-fold) with butyrate, indicating its importance in PMF formation during syntrophic growth.

Both MAGs encoded multiple F-type and V-type ATP synthases, and part of them were expressed and demonstrated upregulation specifically after butyrate addition. The two types of ATPases might translocate sodium and proton, respectively [44], but could also function as PMF or ATP generators in different metabolisms. The other model SBOB only encoded one type of ATPase [7, 46], probably due to their less versatile metabolisms (see Supplementary Notes).

### Propionate oxidation via a Pelotomaculaceae member (F70)

The family Pelotomaculaceae contains several syntrophic propionate oxidizing bacteria (SPOB) [51–54], but as described below, the MAG F70 represented a novel species (Supplementary Notes and Data S6) with a versatile metabolism in addition to the syntrophic lifestyle. We have given it the provisional name *Ca*. Propionivorax syntrophicum F70.

#### Methylmalonyl-CoA pathway

F70 encoded and expressed the conventional propionate oxidation pathway: propionate uptake, activation to propionyl-CoA, carboxylation to methylmalonyl-CoA, isomerization to succinyl-CoA, oxidative decarboxylation to acetyl-CoA, dethiolation to acetate, and acetate export (Fig. 4, Fig. S3, Data S4). To activate propionate, it encoded a CoA ligase to catalyze the exergonic reaction, but also encoded a CoA transferase to couple this reaction with the downstream exergonic acetyl-CoA dethiolation, which can otherwise be independently catalyzed by the acyl-CoA synthetases. All three types of genes showed positive responses to propionate addition. However, one of the acyl-CoA synthetases was upregulated 38-fold, much higher than the others, indicating this population likely decouples propionate activation and acetyl-CoA hydrolysis and does not conserve energy here. F70 encoded and expressed both an ATP-consuming carboxylase and a carboxyltransferase. The carboxyltransferase can couple carboxylation of propionyl-CoA with the downstream oxaloacetate decarboxylation, and the latter step could also be catalyzed by solely an oxaloacetate decarboxylase (Oad) with extrusion of two sodium ions. Therefore, from oxidizing each molecule of propionate, this population could yield one ATP from substrate-level phosphorylation at the succinyl-CoA synthesis step and four reducing equivalents including: one NADH and MKH_2_, and two Fd_red_, each carrying two electrons.

#### H_2_/formate production and electron confurcation/bifurcation mechanisms

The membrane bound succinate dehydrogenases transfer the electrons to MK, and the formed MKH2 can be reoxidized by the membrane-bound cytochrome b-linked quinone-dependent hydrogenases (HydA–HybB) and formate dehydrogenases (FdnGH–HybB), similar to other SPOB of the genus *Pelotomaculum* [51]. F70 also encoded and expressed several other cytoplasmic hydrogenases (HydA, HndCD) and formate dehydrogenases (Fdh), which can consume reducing equivalents of NADH (or NADPH) and Fd_red_ to form H_2_ and formate. The Fdh–Hyl complex is an electron-confurcating formate dehydrogenase capable of driving endergonic NADH oxidation using exergonic oxidation of Fd_red_ [51, 55]. It is suspected to function in this bacterium, as the related genes found in the MAG were specifically upregulated with propionate. The formate produced inside the cytoplasm may be pumped out via a formate transporter (FocA), which was upregulated (7.7-fold) only after propionate addition. Generally, both formate dehydrogenases (such as FdnGH–HybB and Fdh–Hyl) and hydrogenases (like HydA) were upregulated in response to propionate, implying formate and H_2_ may both contribute to interspecies electron transfer between SPOB and methanogens.

#### Other electron transferring proteins indicating sulfate-reducing metabolism

In MAG F70, three complexes (HDR1-3) containing heterodisulfide reductases (Hdr) were encoded and expressed: the first two were highly upregulated after propionate addition, and HDR3 was expressed under all conditions. Similar Hdr-containing complexes have been detected in sulfate-reducing bacteria (SRB) [56] and some syntrophic bacteria, such as *S. fumaroxidans* [57], indicating their potential roles in sulfur metabolism or specialized low-energy metabolisms. In addition, several genes required for dissimilatory sulfate reduction (DSR) were detected in this genome, including sulfate adenylyltransferase (*sat*), a *dsrC* subunit, pyrophosphatase (*hppA*), and putative anaerobic sulfite reductase (*asrAB*), indicating F70 might also play an important function in sulfate-reducing metabolism (SR) under sulfidogenic conditions. The Hdr with the Flx proteins were proposed to couple the reduction of ferredoxin by NAD(P)H with the reduction of DsrC in many SRB [58]. Expression of such related genes by F70 could thus serve as another Fd_red_ source for H_2_ or formate production.

#### PMF and ATP synthesis

Besides substrate-level phosphorylation, ATP could also be produced via an F0F1-type ATP synthase for F70, which was expressed specifically with propionate. PMF could be generated by the proton-translocating HppA, Oad, and possibly the Hyb- and Fdn-containing membrane-bound complexes [51] (see Supplementary Notes).

### Distribution of syntrophic bacterial populations in Danish digesters

**Fig. 5:**
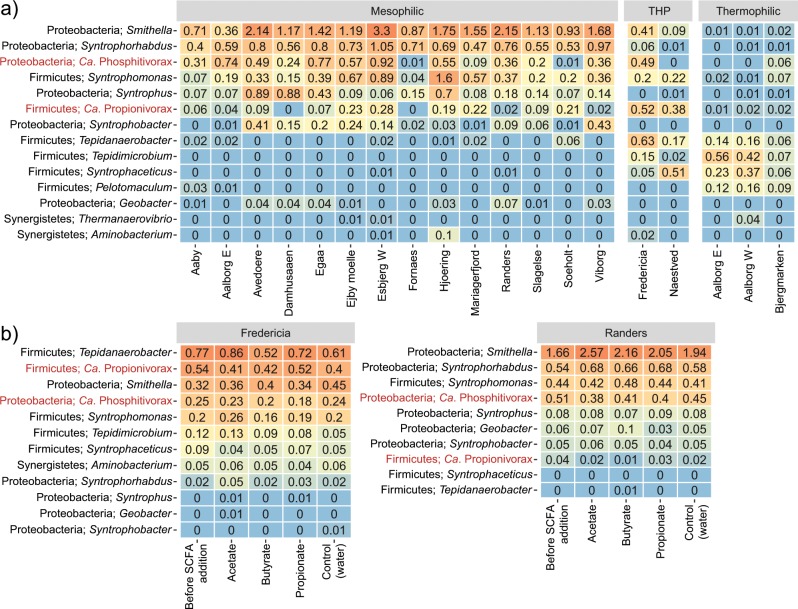
Relative abundance of known and novel syntrophic bacterial genera in (**a**) full-scale digesters and (**b**) the SCFA stimulated reactors. Data are based on 16S rRNA gene amplicon sequencing of DNA extracted from digester slurry. The full-scale data were based upon 103 samples collected from 30 full-scale digesters at 18 Danish WWTPs in 2016 year with 2 to 12 samples per digester [8]. The numbers in the heatmap are average relative read abundance in percentage for each WWTP. The THP digesters are operated at mesophilic conditions, but the substrates are thermally hydrolyzed prior to anaerobic digestion. The lab-scale data were derived from analyses on the inoculum and biomass sampled 2 h after each SCFA addition, with two samples for each condition. Taxonomic classification was based on the SILVA taxonomy. The novel syntrophic genera described in this study are highlighted in red.

A survey into the bacterial composition based on 16S rRNA gene amplicon sequencing was conducted on full-scale anaerobic sludge digesters located at 18 WWTPs in Denmark. As the newly discovered syntrophs are not described in the 16S rRNA reference database used for classification, the 16S rRNA gene sequences associated with the three MAGs were extracted and used as reference to investigate their distribution in sludge digesters (Fig. 5a).

*Ca*. Phosphitivorax was detected in the mesophilic reactors from all 14 plants, although only at low abundance in two plants. It ranks as the third most abundant genus comprising the currently known syntrophs (called “syntrophic genus”) across all plants, suggesting that it is important for the anaerobic digestion process. It was also observed in one of the two investigated THP plants, for which biomass in the digester feedstock has been thermal hydrolyzed before digestion at mesophilic condition. This genus was rarely detected in the thermophilic digesters.

*Ca*. Propionivorax was observed in mesophilic digesters from 12 plants and in both THP plants. This population was also fairly abundant in the full-scale digesters and ranked as the sixth most abundant syntrophic genus (Fig. 5a). Its read abundance was especially high in the THP plant, where it was the second most abundant syntroph. Nevertheless, this population was only observed at very low read abundance in the thermophilic reactors.

Both *Ca*. Phosphitivorax and *Ca*. Propionivorax were not detected in the incoming feedstocks, indicating they originate from and grow in digesters. The broad distribution of these lineages across WWTPs indicates that they may be important but previously overlooked syntrophs in full-scale digesters.

Amplicon analysis of syntrophic bacteria in the lab-scale reactors showed that *Ca*. Phosphitivorax and *Ca*. Propionivorax were in low relative abundances and had no detectable growth (Fig. 5b). A similar phenomenon was observed for other syntrophs (Fig. 5b), as considered to be in agreement with their slow growth rates. However, as conversion of propionate and butyrate started immediately after addition, and intermediates (like formate) did not accumulate, we can thus conclude that even a low number of syntrophs can effectively convert the SCFAs to methanogenic precursors and transfer them to methanogens, as also observed in other methanogenic systems [9, 17, 18].

## Conclusions and future perspectives

Transcriptomic responses of 198 microbial members from two full-scale ADs revealed three novel syntrophic bacteria, including one SPOB of the family Pelotomaculaceae and two SBOB of the genus *Ca*. Phosphitivorax, which were found to be ubiquitous in ADs. Annotation of all three genomes demonstrated common features of syntrophic SCFA-oxidizing bacteria, such as a general beta-oxidation or methylmalonyl-CoA pathway, multiple paralogs for each enzymatic function but selective expression under different environmental conditions, various formate dehydrogenase/hydrogenase and electron bifurcation/confurcation metabolisms. At the same time, they displayed specific characteristics, such as multiple ATPases, NADPH-utilizing electron-transfer system, and versatile metabolisms. *Ca*. Phosphitivorax anaerolimi F81 can catalyze syntrophic butyrate oxidation for energy, and could, based on genomic annotation, probably use isobutyrate, ethanol, and other organics for a heterotrophic lifestyle, but also has the genetic potential for dissimilatory phosphite oxidation and nitrite reduction with CO_2_ fixation. *Ca*. Phosphitivorax butyraticus R76 seems to lack only the phosphite oxidation metabolism compared with F81. *Ca*. Propionivorax syntrophicum F70 demonstrated syntrophic propionate oxidation capability, but also encoded genes for dissimilatory sulfate/sulfite reduction. Genomic features for such chemolithotrophic metabolisms indicated that these three bacteria may not strictly live a syntrophic lifestyle, but could also survive via versatile metabolisms, which need to be verified in further studies. The in situ activities of these low-abundant microbial members can be effectively detected by the genome-guided metatranscriptomic approach, demonstrating the high sensitivity of this method in studying the behavior of individual members in complex microbiomes. Nevertheless, future improved sequencing technologies and genome construction tools are expected to extend the range of microbial members studied by this approach and increase the efficiency.

## Supplementary information

Supplementary information

Supplementary Data S1

Supplementary Data S2

Supplementary Data S3

Supplementary Data S4

Supplementary Data S5

Supplementary Data S6

## Data Availability

All sequencing data have been submitted to European Nucleotide Archive under the project ID PRJEB31310. Accession numbers for individual dataset can be found in Data S1.
