# Supplementary material for: Novel syntrophic bacteria in full-scale anaerobic digesters revealed by genome-centric metatranscriptomics
Source: ISME J. 2020 Jan 2;14(4):906–18. doi: 10.1038/s41396-019-0571-0 (PMC7082340; doi:10.1038/s41396-019-0571-0)
Supplement: Supplementary file 1 — Supplementary information [file 41396_2019_571_MOESM1_ESM.pdf]

Supplementary Information

Novel syntrophic bacteria in full-scale anaerobic digesters revealed by genome-centric metatranscriptomics

Authors: Liping Hao[1,2], Thomas Yssing Michaelsen[1], Caitlin Margaret Singleton[1], Giulia Dottorini[1], Rasmus Hansen Kirkegaard[1], Mads Albertsen[1], **Per Halkjær Nielsen[1*], Morten Simonsen Dueholm[1*]**

Affiliation:
[1]Center for Microbial Communities, Department of Chemistry and Bioscience, Aalborg University, Aalborg, Denmark.
[2]State Key Laboratory of Pollution Control and Resource Reuse, College of Environmental Science and Engineering, Tongji University, Shanghai, P.R. China.

Supplementary Methods

1.1 Chemical analyses

Short-chain fatty acid (SCFA) analysis. The preserved digester slurry samples were thawed on ice, and centrifuged at 14 000 x g for 5 min at 4°C to separate the supernatant, which was diluted and filtered through 0.22 μm pore size filters. The filtrate was used for analyzing the concentrations of SCFAs. SCFAs were analyzed with a High-Pressure Ion Chromatography (HPIC, Dionex ICS-5000 system, Thermo Scientific™), equipped with an IonPac AS11-HC capillary column and a conductivity detector. The autosampler tray temperature was set to 10°C; the sample volume injected into the system was 25 μl; column was operated at 30°C. Ultrapure water and potassium hydroxide (60 mM) were used as eluents for a multi-step gradient elution. Eluents were used with flow-rate of 1.5 mL/min. A total HPIC program took 37.5 min per sample. Potassium hydroxide concentration was 1 mM at the start of the run for the first 13 min; then it was increased steadily to 3 mM until 26 min; subsequently, the concentration was raised to 60 mM at 28 min and kept at this value for 5 min, and then decreased to 1 mM until the end of the program. With this program, nine organic acids commonly produced in anaerobic digestion processes could be analyzed, including: formate, acetate, propionate, butyrate, isobutyrate, valerate, isovalerate, lactate, and hexanoate, and the detection limit was 10 μM.

Methane production. Methane yield from all the reactors during the 3-day incubation was continuously recorded by the AMPTS II, for which the volume was corrected to standard conditions (20°C, 1 atm). Net methane production from the added SCFAs was calculated by subtracting the average total methane yield of the control reactors from that of the reactors stimulated with SCFAs.

1.2 Metagenomic analyses

Two slurry samples from Fredericia (indicated by F-) and five from Randers (indicated by R-) were used for metagenomics studies. F1 and R1 were taken from the SCFA stimulation experiment; F2 and R2 were taken from the full-scale digesters 2 months before the SCFA stimulation study; R3, 4, 5 derived from the full-scale digester at earlier different time points (**Data S1**).

DNA extraction. DNA used for sequencing was extracted using the FastDNA Spin kit for soil (MP Biomedicals, Santa Ana, CA, USA) as previously described (Kirkegaard *et al.*, 2016). For each of F1, 2 and R1, 2, DNA was extracted at two different bead-beating intensities (**Protocol 1**: 4 m/s for 20 s; **Protocol 2**: 4 times of 6 m/s for 40 s), providing 8 metagenomes in total (two for each sample) based on different extraction efficiencies to facilitate the binning of MAGs.

Metagenomic Sequencing. Metagenome libraries were prepared with the Illumina Nextera DNA Library Prep kit. The libraries were paired-end (2 x 250 bp) sequenced on the Illumina HiSeq 2500 platform using the HiSeq Rapid PE cluster kit v2 and the HiSeq Rapid SBS kit v2 (500 cycles) in rapid run mode and also paired-end sequenced (2 x 300 bp) on Illumina MiSeq platform using MiSeq reagent v3 (600 cycles). Standard protocols were used for sample preparation and sequencing. 3 sequencing events on different dates with the DNA samples mentioned above generated 12 DNA sequencing datasets, which were the 12 metagenomes (as shown in Data S1).

Assembly. Raw paired-end FASTQ data were imported into CLC Genomics Workbench v. 9.5.2 (QIAGEN Bioinformatics) and processed. The paired-end reads were trimmed using the Trim Sequences tool (phred ≥20 corresponding to quality limit 0.01, min length 50 bp, no ambiguous bases and removal of Nextera adaptor sequences). Metagenome assembly was performed using the De Novo Assembly tool (word size 64, automatic bubble size, min scaffold length 1000 bp, auto-detect paired distances, perform scaffolding) for 12 single samples and three sample groups (according to their origin and sampling time), resulting in 12 single-assemblies and six co-assemblies (**Table S2**). Two co-assembly settings were used: for one set, the reads of broken pairs separated during trimming were used for assembly, and mapping was set as: length 1.0, similarity 0.95; for another set, broken pairs were not used, and mapping was set as: length 0.7, similarity 0.95. Assembly read coverage was estimated individually by mapping each library to the metagenome assembly using the "Map Reads to Reference" tool.

Phylogenetic tree of the genomes. The phylogenetic genome tree of the bacterial MAGs was created using GTDBtk v0.1.3 classify_wf and the associated RefSeq release 86 following the default parameters (Parks *et al.*, 2018). MAG genes were called using Prodigal v2.6.2 (Hyatt *et al.*, 2010), and marker genes were identified using HMMER v3.1 (Eddy, 2011). 120 single copy marker genes were concatenated and aligned, and pplacer v1.1 (Matsen et al., 2010) was used to determine the MAG placement in the overall bootstrapped GTDB tree. Bootstrap values shown are from the original GTDB tree. The trees were visualized and pruned in ARB (Ludwig et al., 2004) to show only the MAGs recovered in this study. iTOL (Letunic and Bork, 2016) and Inkscape (https://inkscape.org/) were used for further refinements.

1.3 Genome-centric metatranscriptomic analyses

30 samples in total (15 from each digester) were used for metatranscriptomic analysis (**Data S1**). These included 3 biological replicates from one of the reactors at each condition, sampled 1 hour after addition of acetate, propionate, butyrate, or water (as control), respectively, and 1 hour before SCFA addition (from the same reactor for butyrate addition, as all reactors were operated at the same condition at that moment).

RNA extraction and purification. Prior to RNA extraction, samples (0.5 mL) were thawed on ice, and centrifuged for 10 min at 14 000 g at 4°C to remove the liquid phase and collect cell pellets (about 0.1 g). Total RNA was extracted using the RNeasy PowerMicrobiome Kit (Qiagen) following the manufacturer's instruction with the addition of phenol:chloroform:isoamy alcohol (25:25:1) and β-mercaptoethanol (10 µl/mL final concentration). Bead-beating (40 s at 6 m/s, four times with two min interval on ice) on a Fastprep FP120 (MP Biomedicals) was performed for cell lysis instead of vortexing. The total RNA extracts were subjected to DNase treatment to remove DNA contaminants by using the DNase Max Kit (Qiagen), and further cleaned up and concentrated with the Agencourt AMpure XP magnetic beads (Beckman Coulter) before rRNA depletion.

The integrity and quality of the purified total RNA were assessed on a Tapestation 2200 (Agilent, California, USA) with the Agilent RNA screen-tapes (Agilent, USA), and the concentration was

measured using Qubit RNA HS Assay Kit (Thermo Scientific Fisher, USA). The average RNA Integrity Number (RIN) was 5.2 and 6.0 for samples from Randers and Fredericia, respectively.

rRNA depletion, library preparation and sequencing. For all 30 samples, rRNA was depleted from the total RNA using the Ribo-Zero rRNA Removal (Bacteria) Kit (Illumina, San Diego, CA, USA), with two µg total RNA as input. The quality of extracted mRNA was checked using Agilent RNA HS screen-tapes (Agilent, USA) and the concentration was determined by Qubit RNA HS Assay Kit (Thermo Scientific Fisher, USA). The TruSeq Stranded mRNA Sample Preparation Kit (Illumina; San Diego, CA, USA) was used to prepare cDNA sequencing libraries according to the manufacturer's instruction. The prepared libraries were sequenced on an Illumina HiSeq2500 using the TruSeq PE Cluster Kit v3-cBot-HS and TruSeq SBS kit v.3-HS sequencing kit (1x50 bp; Illumina Inc.).

Trimming and detection of rRNA reads. Raw RNA reads in FASTQ format were imported into CLC Genomics Workbench v. 9.5.2 and trimmed to remove adaptors and for quality, requiring a minimum phred score of 20 and a read length of 45. The rRNA reads were then filtered by mapping the trimmed RNA reads to a Prokaryotic 16S and 23S rRNA sequence database which was generated from the SILVA_132_SSURef and SILVA_132_LSURef databases (Quast *et al.*, 2013; Yilmaz *et al.*, 2014), with a minimum similarity of 98% over 80% of the read length, and the unmapped were considered as the mRNA sequences.

Calculation of relative abundance of MAGs in metagenome and metatranscriptome. The numbers of DNA or mRNA reads mapped to the contigs or CDSs of each MAG were calculated, and normalized to the read number size of the metagenome or metatranscriptome datasets, which were presented in **Data S3** as relative **percentage** abundance.

Besides, in order to gain a library- and genome-size independent calculation of the MAG abundance and MAG transcription, Reads Per Kb-MAG/CDSs per Million reads (RPKM) were calculated for each genome and transcriptome. The ratio of RNA_RPKM to DNA_RPKM provides a standardized calculation of activity for each MAG. These data were also provided in **Data S3**.

1.4 Evaluating the distribution of interesting members by amplicon sequencing

Extraction of 16S rRNA gene sequences from interesting MAGs. Nearly full-length 16S rRNA gene sequences associated with the MAGs were extracted according to Open Reading Frame (ORF) calling and annotation with Prokka (v1.12) (Seemann, 2014) or identified according to the link between contigs by using mmgenome2 (https://github.com/KasperSkytte/mmgenome2).

Amplicon sequencing analysis. Raw amplicon sequencing data by using samples from Danish anaerobic digesters at WWTPs taken in 2016 year was obtained from Kirkegaard *et al.*, (2017). Forward reads of the V1-3 amplicons were concatenated and processed using usearch v.10. 0.240 (Edgar, 2010). Raw fastq files were filtered for phiX sequences using -filter_phix, trimmed to 250 bp using -fastx_truncate -trunclen 250, and quality filtered using -fastq_filter with -fastq_maxee 1.0. The

sequences were dereplicated using -fastx_uniques with -sizeout -relabel Uniq. Exact amplicon sequence variants (ASVs) were generated using -unoise3 (Edgar, 2016). ASV-tables were created by mapping the raw reads to the ASVs using -otutab with the -zotus and -strand both options. Taxonomy was assigned to ASVs using -sintax with -strand both and -sintax_cutoff 0.8 (Edgar, 2018) with the MiDAS 3.5 reference database (Nierychlo et al., (manuscript in preparation)). Amplicons were mapped to full-length 16S rRNA gene sequences associated with the three MAGs using **-usearch_global** with **-id 0.945 -strand both** to link ASVs with MAGs (Edgar, 2010). The data was further analyzed in R (R Core Team, 2017) using Ampvis2 (Andersen *et al.*, 2018).

1.5 Comparison of genomes and 16S rRNA gene sequences

The genomes showing highest similarity with MAGs F70, F81, and R76 were identified from the Genome Taxonomy DataBase (GTDB) (release 03-RS86) using GTDB-Tk (v0.1.3) and the GTDB-Tk classify workflow (Parks *et al.*, 2018).

Identification of protein homologs between MAGs F70, R76, F81 and their closest relatives. Annotated protein sequences were obtained directly from the Microscope platform for F70, R76 and F81, and from the IMG platform for *Candidatus* Phosphitivorax anaerolimi strain Phox-21 (IMG genome ID 2657245163). MAGs were obtained as unclassified nucleotide sequences from the genome taxonomy database for *Pelotomaculum* sp. UBA1371 (GCA_002305915.1) and Syntrophaceae bacterium UBA1062 (GCA_002316295.1) and classified using EnrichM (https://github.com/geronimp/enrichM). Protein sequences from each genome was blasted against those from other genomes using usearch11 with the ublast tool (usearch -ublast query.fa -db target.fa -maxrejects 0 -maxaccepts 0 -top_hit_only -evalue 1e-9 -blast6out query_vs_target.b6. Homologs were identified based on the e-value cutoff. The results are shown in **Data S5**.

Genome comparison. According to the GenBank assembly accession of the 3 closest relatives, genome files were downloaded from GenBank. Besides, genomes files of several known syntrophic SCFA-oxidizing bacteria were obtained from GenBank, the JGI genomes online database, the internal JSpeciesWS genome database (Version: 3.0.20) or directly acquired from the authors (e.g., *Pelotomaculum schinkii* HH) (Hidalgo-ahumada *et al.*, 2018). These genomes were then compared using JSpeciesWS by analyzing the average nucleotide identity (ANI) based on BLAST+(ANIb) calculation (Richter *et al.*, 2015). The results are shown in **Data S6**.

16S rRNA gene comparison. The 16S rRNA gene sequences associated with the three MAGs were used as query sequences in NCBI Standard Nucleotide BLAST, to search for the closest relatives.

Supplementary Notes

2.1 The three newly-proposed syntrophs and their closest relatives in the public databases

The closest relatives of MAGs F70, F81, and R76 were identified by blasting their genomes and 16S rRNA gene sequences in GTDB and NCBI nucleotide database. These genomes, together with those of the known syntrophs, were further compared to search for similarities and differences, as described below.

Phylogenetic position of F70, a member of the family Pelotomaculaceae

F70, a 2 Mbp draft genome (completeness 74.7%; contamination 2.5%) has an ANI of 70.99% and alignment of 40.53% with the 3 Mbp genome of *Pelotomaculum thermopropionicum* SI (Kosaka *et al.*, 2008), which is a thermophilic, syntrophic propionate-oxidizing bacterium (SPOB) (Imachi *et al.*, 2002). The genome alignment result is similar (71.14% ANI and 44.48% alignment) with another known SPOB *Pelotomaculum schinkii* HH (Hidalgo-ahumada *et al.*, 2018). Based on GTDB, the closest (98.61% ANI and 79.72% alignment) MAG of F70 is GCA_002305915 (completeness 75.44%, contamination 0%), which is classified to: Bacteria; Firmicutes_B; Desulfotomaculia; Desulfotomaculales; Pelotomaculaceae; DTU098 (and the corresponding NCBI taxonomy: Bacteria; Terrabacteria group; Firmicutes; Clostridia; Clostridiales; Peptococcaceae; Pelotomaculum; Pelotomaculum sp. UBA1371) (Parks *et al.*, 2017). It is assembled from a metagenome constructed from an anaerobic digester treating palm oil mill effluent (ERR276848) (Bala *et al.*, 2014). Similarly, the other MAGs in the same genus (DTU098) all originate from anaerobic digesters treating different types of organic wastes (Campanaro *et al.*, 2016; Parks *et al.*, 2017).

A 1535-bp long 16S rRNA gene sequence extracted from the refined draft genome showed 99% identity with an uncultured bacterium clone DangY118-A12 (KC333938.1), and 94% identity with *Cryptanaerobacter phenolicus* LR7.2 T (NR_025757.1) and *Pelotomaculum* sp. FP (AB159558.1). The former is an anaerobic bacterium that transforms phenol and 4-hydroxybenzoate into benzoate (Juteau *et al.*, 2005), while the latter is a mesophilic, spore-forming SPOB (de Bok *et al.*, 2005). The 16S rRNA gene of F70 also demonstrated similarity lower than 95% with the known SPOB like *Pelotomaculum thermopropionicum* SI, *Pelotomaculum schinkii* HH, and *Pelotomaculum propionicicum* strain MGP (Hidalgo-ahumada *et al.*, 2018).

The difference in genome ANI combined with phylogenetic analysis indicates that F70 is likely a new genus of the family Pelotomaculaceae (based on MAG placement in GTDB) (Jain *et al.*, 2018), or Peptococcaceae (based on the 16S rRNA gene sequence in NCBI taxonomy).

Phylogenetic position of F81 and R76, two members of class Desulfomonilia

F81 and R76 were both classified to Bacteria; Desulfobacterota; Desulfomonilia; UBA1062; UBA1062; UBA1062, but their genomes illustrated some difference (with 78.32% ANI and 64.22% alignment), and have greater similarity to different public genomes, indicating classification to different species (Konstantinidis and Tiedje, 2005; Richter *et al.*, 2015; Jain *et al.*, 2018). Two 1562-

bp long 16S rRNA gene sequences, were retrieved from the two genomes respectively, which showed 98% identity with each other.

F81, a 3.2 Mbp MAG (completeness 95.7%; contamination 1.3%) demonstrated highest similarity (99.21% ANI and 93.8% alignment) to *Candidatus* Phosphitivorax anaerolimi strain Phox-21 (phylum Desulfobacterota; class Desulfomonilia; order UBA1062; family UBA1062; genus UBA1062; species GCA_001896555.1, completeness 98.49%; contamination 1.94%). Further, the 16S rRNA gene sequence of F81 also showed 99% identity with that of strain Phox-21 (1541 bp, KU898264.1), indicating they are within the same species.

R76, a 3 Mbp MAG (completeness 86.86%; contamination 0.38%) was closest (99.03% ANI and 84.63% alignment) to Syntrophaceae bacterium UBA1062 (phylum Desulfobacterota; class Desulfomonilia; order UBA1062; family UBA1062; genus UBA1062; species GCA_002316295.1, completeness 86.9%; contamination 2.1%), which is assembled from a metagenome (SRX371701) of anaerobic digester sludge. The 16S rRNA gene of R76 shared the highest similarity (98% identity with 97% alignment) with an uncultured Deltaproteobacteria clone 81 (1527 bp, GU112205.1) originating from biogas slurry derived from anaerobic fermentation of pig manure, but has only 97% similarity (with 99% alignment) with strain Phox-21, indicating they could be assigned to different species within the same genus *Ca.* Phosphitivorax.

Comparative genomic analysis indicating similar major metabolisms

The comparative genomic analysis between the MAGs F70, F81, R76 and their closest relatives was shown in **Data S5**. The results revealed no apparent differences in genetic potential for major metabolisms, which suggests that the observations made in this study can be extrapolated to the close relatives.

The comparative genomic analysis for F81 and R76 showed that, 2477 out of 3223 proteins encoded by F81 have homologs in R76 (**Data S5**). These included the enzymes involved in the metabolisms such as butyrate beta-oxidation, inorganic carbon fixation via the reductive glycine pathway, nitrite oxidation, formate/hydrogen production, electron bi/confurcation, etc. These shared genes also demonstrated quite similar transcriptomic behavior under SCFAs and Control conditions, indicating high similarity in major metabolisms. Nevertheless, enzymes responsible for phosphite oxidation are unique to F81, while a few proteins involved in glycerol degradation only appeared in R76, indicating varied accessory metabolisms for these two species. However, the lack of such “unique” genes could also be due to the incompleteness of the MAGs.

2.2 Other bacterial and archaeal MAGs showing responses to SCFA stimuli

Bacterial MAGs F82 and R77, R51 expressed genes for most steps in propionate or butyrate oxidation, respectively (**Figure 3, Figure S2**), which could be potential propionate or butyrate oxidizers. The first was assigned to Anaerolineaceae, indicating it could be a potential novel SPOB. The latter two were classified to the order Syntrophales, similar as the model SBOB *Syntrophus aciditrophicus,* implying they could be potential SBOB and phylogenetically related to the model ones. However, in

these MAGs, the SCFA-oxidation associated genes were not upregulated as much as the member F70 (for propionate), and the member F81 (for butyrate). F82 demonstrated high similarity with *Brevefilum fermentans*, which encoded the genes associated with the methylmalonyl-CoA pathway. This bacterium was previously proposed to catalyze propionate production from fermentation of amino acids, thus using the pathway in the opposite direction (McIlroy *et al.*, 2017). However, the current data indicated that this may not always be the case.

Syntrophic cooperation between SCFA-oxidizing bacteria and archaea was indicated by the transcriptomic behavior of the archaeal members. The archaeal populations contributed to a high percentage (for instance, 3.02% for F94) of all transcripts, indicating high methanogenic activities in these systems. All acetotrophic methanogens (genus *Methanothrix*) demonstrated higher activities after acetate and butyrate addition [for instance, the $mRNA_{-RPKM}/DNA_{-RPKM}$ ratio for MAG R103 increased from 13 (before) to 39 (acetate) and 25 (butyrate), and such ratio numbers are 43 (before), 79 (acetate) and 62 (butyrate) for MAG R97; and 11 (before), 17 (acetate) and 13 (butyrate) for MAG F94]. They were the major members responding to the acetate directly added or produced from oxidation of SCFAs with longer carbon chain; while several H_2- and formate-utilizing methanogens (genus *Methanoculleus*, *Methanospirillum*, and family Methanoregulaceae) demonstrated positive responses to propionate and butyrate addition [for instance, the $mRNA_{-RPKM}/DNA_{-RPKM}$ ratio for MAG R99 increased from 12 (before) to 18 (propionate) and 26 (butyrate), and such ratio numbers are 17 (before), 27 (acetate) and 25 (butyrate) for MAG R98] (**Data S3**). This suggests that more acetate, H_2, and formate were produced after the addition of SCFAs. These products were likely used for interspecies electron transfer (IET) between the syntrophic SCFA-oxidizing bacteria and the cooperating methanogens, similar to the frequently described relationship of the model syntrophs (Müller et al., 2010; Stams and Plugge, 2009; Kouzuma et al., 2015).

2.3 Other details in metabolic pathway reconstruction for F70

Methylmalonyl–CoA pathway. The oxaloacetate decarboxylation step can also be performed via a membrane-bound oxaloacetate decarboxylase complex, which extrudes two sodium ions out of the cell while decarboxylating oxaloacetate to pyruvate with proton motive force (PMF) produced, but genes encoding this complex were not highly expressed at any of the studied conditions (**Data S4**).

H_2/formate production and electron confurcation/bifurcation mechanisms. The formate dehydrogenases (FdnGH-HybB) contain a twin-arginine translocation (Tat) pathway conserved site, which points to the translocation of these proteins across the cytoplasmic membrane (Sedano-Núñez *et al.*, 2018), indicating production of formate at the outside of the cytoplasmic membrane (Stams and Plugge, 2009). For the membrane-bound hydrogenases (HydA-HybB), it is not clear on which side of the membrane they are located.

The Fdh-Hyl enzyme complex is an electron-confurcating formate dehydrogenase capable of driving endergonic formate-generating NADH ($E°'$ = -230 mV) oxidation using exergonic formate-generating oxidation of Fd_{red} ($E°'$ = -430 mV) (Wang *et al.*, 2013; Hidalgo-ahumada *et al.*, 2018). It is suspected to also exist in F70, as a few of the components (in FDH4) were found in the draft

genome (located on different contigs due to poor genome quality) and expressed, such as the formate dehydrogenase *fdhF2* with active-site selenocysteine and molybdopterin, the proteins involved in molybdopterin cofactor synthesis (MogA, MoaC, MobB), and a rubredoxin (Rbr) (Wang *et al.*, 2013).

Other electron transferring proteins. F70 encoded an acyl-CoA dehydrogenase-electron transfer flavoprotein complex (AcdB-FixAB), similar to that encoded by *S. fumaroxidans* (Sedano-Núñez *et al.*, 2018). This complex was specifically upregulated under propionate addition, indicating its potential role in propionyl-CoA conversion in the syntrophic propionate oxidation process.

The predicted function of heterodisulfide reductase (Hdr). F70 encoded three complexes (HDR1-3) containing heterodisulfide reductases (Hdr): in HDR1, the heterodisulfide reductases are clustered with two methyl-viologen-reducing hydrogenase subunits (MvhBD) and an anaerobic sulfite reductase subunit (AsrA); HDR2 only contains HdrA, B, C, D subunits; HDR3 was again a multicomplex that includes a sulfur carrier protein required for formate dehydrogenase activity (FdhD), a MvhD and an Fe-S oxidoreductase. And in HDR1 and HDR2, several subunits are putatively associated with flxDCBA, indicating that the Flx complex could also exist for this microbial member.

The heterodisulfide reductase (Hdr) present in methanogens performs the reduction of CoM-S-S-CoB heterodisulfide to CoM-SH and CoB-SH (Hedderich *et al.*, 2005). This enzyme was also detected in sulfate reducing bacteria (SRB) (Grein *et al.*, 2013) and some syntrophic bacteria, like the aromatic compound-degrading *Syntrophorhabdus aromaticivorans* (Nobu *et al.*, 2015a), butyrate-oxidizing *Syntrophomonas wolfei* (Sieber *et al.*, 2015), and propionate-oxidizing *S. fumaroxidans* (Sedano-Núñez *et al.*, 2018). In these bacteria, such Hdr-containing complexes were thought to facilitate electron confurcating hydrogen/formate production with Fd_{red}/NADH/F420 oxidation coupled with reduction of heterodisulfide or DsrC (Buckel and Thauer, 2013; Nobu *et al.*, 2015b; Buckel and Thauer, 2018; Nobu *et al.*, 2015a; Sedano-Núñez *et al.*, 2018; Sieber *et al.*, 2015; Ramos *et al.*, 2015). Whether the Hdr-like enzymes are involved in syntrophic propionate oxidation (SPO) or sulfate/sulfite reduction (SR) metabolisms is not clear and requires further investigation.

PMF and ATP synthesis. PMF may also be generated by membrane-bound enzyme complexes. For instance, Hyb- and Fdn-mediated menaquinol re-oxidation, as seen for *Pelotomaculum* spp. could potentially release protons on the periplasmic side and contribute to PMF formation (Hidalgo-ahumada *et al.*, 2018). Membrane-bound cytochrome b subunits (i.e., HybB and FdnI) were also found in the F70 genome, demonstrating high similarity (70-80% identity over 95-99% coverage) with the ones found in *Pelotomaculum schinkii,* and indicated that they could be other potential PMF generators. However, translocation of protons during menaquinol re-oxidation step could also be linked to succinate oxidation. Succinate oxidation is a thermodynamically unfavorable reaction. It has been generally accepted that a proton gradient across the membrane is needed, which is produced by the hydrolysis of two-thirds of a molecule of ATP for reverse electron transport (RET). In this process, coupling of the electron transfer via menaquinone/menaquinol with the inward movement of protons, catalyzes the production of formate (and/or) H_2 outside the cytoplasmic membrane (Stams

and Plugge, 2009). Therefore, whether the Hyb- and Fdn-containing enzyme complexes function as PMF generator or consumer need to be further verified.

The ATP synthase could also function in reverse to hydrolyze ATP and export protons, thereby driving the PMF-requiring reactions like succinate oxidation or rotation of flagellum and pili.

Summary. As seen from the genomic and transcriptomic analyses, the F70-represented population demonstrated common features of SPOB. Although it is more phylogenetically close to the *Pelotomaculum* genus in the phylum Firmicutes, from the SPO and SR metabolic point of view, it has greater similarity with *S. fumaroxidans* in the phylum Proteobacteria, which shows the novelty of this newly discovered SPOB in anaerobic digesters.

2.4 Other details in metabolic pathway reconstruction for F81 and R76

Acetone production. Except for production of acetate, the acetyl-CoA moieties generated from acetoacetyl-CoA can also be converted to acetone with an acetoacetate decarboxylase. The formation of final products (acetate or acetone) could be influenced by the surrounding pH, similar to *Clostridium acetobutylicum* (Nölling *et al.*, 2001). Although both types of genes were expressed, one of the acetyl-CoA synthetases became significantly upregulated after butyrate and propionate addition, but was silent before and under acetate and control conditions, indicating activation of this gene by butyrate, and the predominance of an acetate-forming pathway (**Figure S3**).

Butyrate isomerization and PHA metabolism. Interestingly, two vitamin B12-dependent isobutyryl-CoA mutases were expressed with butyrate, which should play important roles in the transformation between butyrate and iso-butyrate (Cracan and Banerjee, 2012; Narihiro *et al.*, 2016; Matihiest *et al.*, 1992), indicating this bacterium might be able to use both isomers as substrates. Similar functions were found for the butyrate-degrading sulfate reducers like *Desulforhabdus amnigenus* (Oude Elferink *et al.*, 1996), and one of the SBOBs *Syntrophothermus lipocalidus* (Sekiguchi *et al.*, 2000; Djao *et al.*, 2010; Narihiro *et al.*, 2016), but not for the model SBOBs like *Syntrophomonas wolfei* (McInerney *et al.*, 1981) and *Syntrophus aciditrophicus* (Jackson *et al.*, 1999). Genes involved in poly-β-hydroxyalkanoate (PHA) metabolism were encoded in the genome, but was nearly not expressed under the studied conditions.

H_2/formate production. Genes involved in the twin-arginine translocation (Tat) pathway and formate dehydrogenases were clustered together, and several formate dehydrogenases (like the *fdoIGH-fdhA*) contain a Tat pathway conserved site, that supported production of formate at the outside of the cytoplasmic membrane. This process was accompanied with inward ion translocation and PMF consumption (Sieber *et al.*, 2010; Crable *et al.*, 2016). Even though this type of formate dehydrogenase was predicted to oxidize formate coupled with nitrate/nitrite reduction by the ammonifying periplasmic nitrite reductase (NrfAH) (Figueroa *et al.*, 2018), the latter gene cluster, as also encoded in the MAGs F81 and R76, was not active under any condition in this study. It further supports MKH2 produced from butyrate beta-oxidation was used by such formate dehydrogenases to generate formate.

Other confurcation/bifurcation mechanisms. Although only NADH was produced from butyrate oxidation, other electron carriers including NADPH and Fd_{red} could be produced from NADH via electron confurcation/bifurcation mechanisms, such as the *Rhodobacter* nitrogen fixation (Rnf) and NAD-dependent ferredoxin:NADPH oxidoreductase (Nfn) complexes encoded by F81 and R76. The ion-translocating Rnf complex could use the ion gradient to drive the unfavourable reduction of ferredoxin by NADH and has been implicated in reverse electron transport (RET) in *S. aciditrophicus* (McInerney *et al.*, 2007). The Nfn is an electron-bifurcating complex that catalyzes the reduction of two $NADP^+$ molecules coupled to the oxidation of reduced ferredoxin and NADH in a reversible manner (Buckel and Thauer, 2013). Both complexes were found for strain Phox-21 and predicted to play key roles for consumption or production of PMF or reducing equivalents in the proposed dissimilatory phosphite oxidation with CO_2 reduction metabolic model (Figueroa *et al.*, 2018). However, they were not highly expressed under current studied conditions, indicating that different metabolisms were active under different environments. Neither complex was reported for the model SBOB *S. wolfei* (Sieber *et al.*, 2010).

Respiration metabolisms. Except for dissimilatory nitrite reduction, F81 and R76 lack the genes for other aerobic and anaerobic respiration (dissimilatory sulfate reduction, dissimilatory nitrate reduction, denitrifying nitrite reduction, or anaerobic ammonium oxidation) as also reported by (Figueroa *et al.*, 2018).

Incomplete Wood–Ljungdahl pathway. The Wood–Ljungdahl pathway (WLP) was described to be used for syntrophic acetate oxidation (SAO) (Müller *et al.*, 2013) or CO_2-fixation in the reverse direction (Ragsdale and Pierce, 2009). All three MAGs encoded an incomplete Wood–Ljungdahl pathway (**Figure S3**, **Data S4**). F70 lacked the genes CO-methylating acetyl-CoA synthase (*acsB*) in the carbonyl branch and methylene-tetrahydrofolate (THF) reductase (*metF*) in the methyl branch, but the rest of the pathway demonstrated similarity with *Desulfotomaculum kuznetsovii*, a rather versatile bacterium of the same order (Desulfotomaculales) which can grow heterotrophically with a large variety of organic substrates (including short-chain and long-chain fatty acids) or autotrophically with $H_2 + CO_2$ and sulfate (Visser *et al.*, 2013). F81 and R76 missed the genes carbon monoxide dehydrogenase (*acsA*) and methyl-THF:CFeSP methyltransferase (*acsE*) in the carbonyl branch, similar as strain Phox-21 (Figueroa *et al.*, 2018).

However, all three MAGs have genes for the NADH (or NADPH)-binding formate dehydrogenase, formate:THF ligase (*fhs*) and methenyl-THF cyclohydrolase/methylene-THF dehydrogenase (*folD*), which can catalyze CO_2 assimilation together with the reductive glycine pathway (RGP) (Bar-Even *et al.*, 2013; Bar-Even, 2016). F81 and R76 have the genes involved in this pathway (*gcvP*, glycine dehydrogenase; *gcvT*, aminomethyltransferase; *gcvH*, lipoate-binding protein*;* *lpd*, dihydrolipoyl dehydrogenase; *glyA*, serine hydroxymethyltransferase; and *tdcB*, threonine and serine deaminase), which were all lacking in F70. Based on such genomic features, it could be predicted that F81 and R76 might conduct autotrophic growth when phosphite and CO_2 are abundant in the environment, whereby dissimilatory phosphite oxidation drives CO_2 reduction to formate, which is then assimilated into biomass via RGP, as described for strain Phox-21 (Figueroa *et al.*, 2018).

The WLP-related genes in F70 were only expressed and highly upregulated (especially the *folD* and *fhs*) with propionate, but were hardly expressed with acetate, indicating that they might not function for SAO, but were probably involved in formate utilization which was produced from SPO. In contrast, the WLP-and RGP-related genes in F81 and R76 were not highly expressed under any condition, except for one acetyl-CoA synthetase (*acsE*) which was also involved in SBO. Thus, the assimilation of inorganic carbon might be inactive when sufficient butyrate is available.

Supplementary Figures

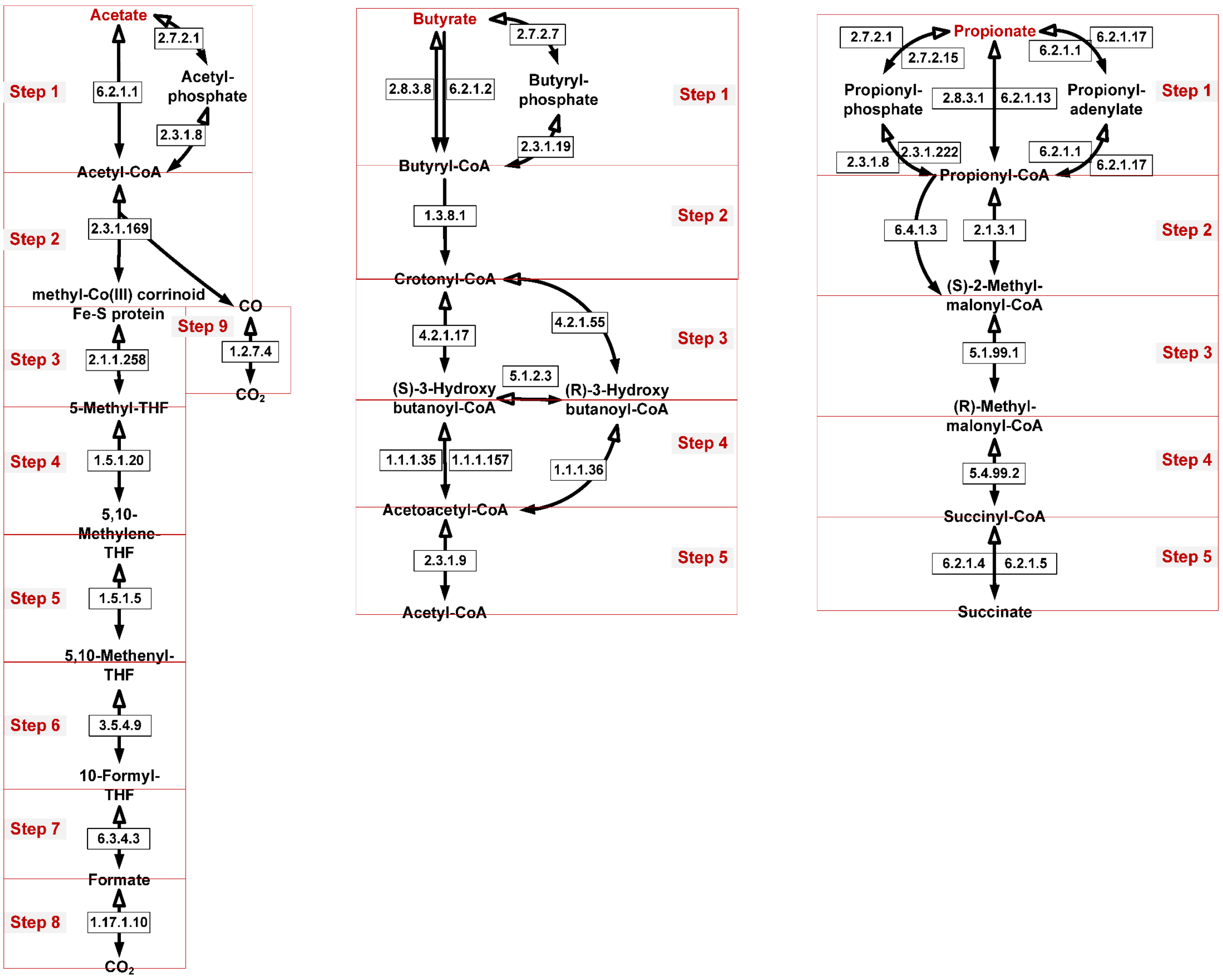

Figure S1. The enzymes directly involved in oxidation of acetate, butyrate, and propionate selected for transcriptomic studies**.** KEGG ontology (KO) numbers associated with these EC numbers are collected as the key genes as shown in **Data S2**. The bioconversion steps meant the major reactions for converting one substance to another of different chemical structure. 13, 19, and 31 gene families determined by KO numbers were used, representing the key genes directly involved in the aforementioned pathways, which can catalyze bioconversion in 9, 5, and 5 steps.

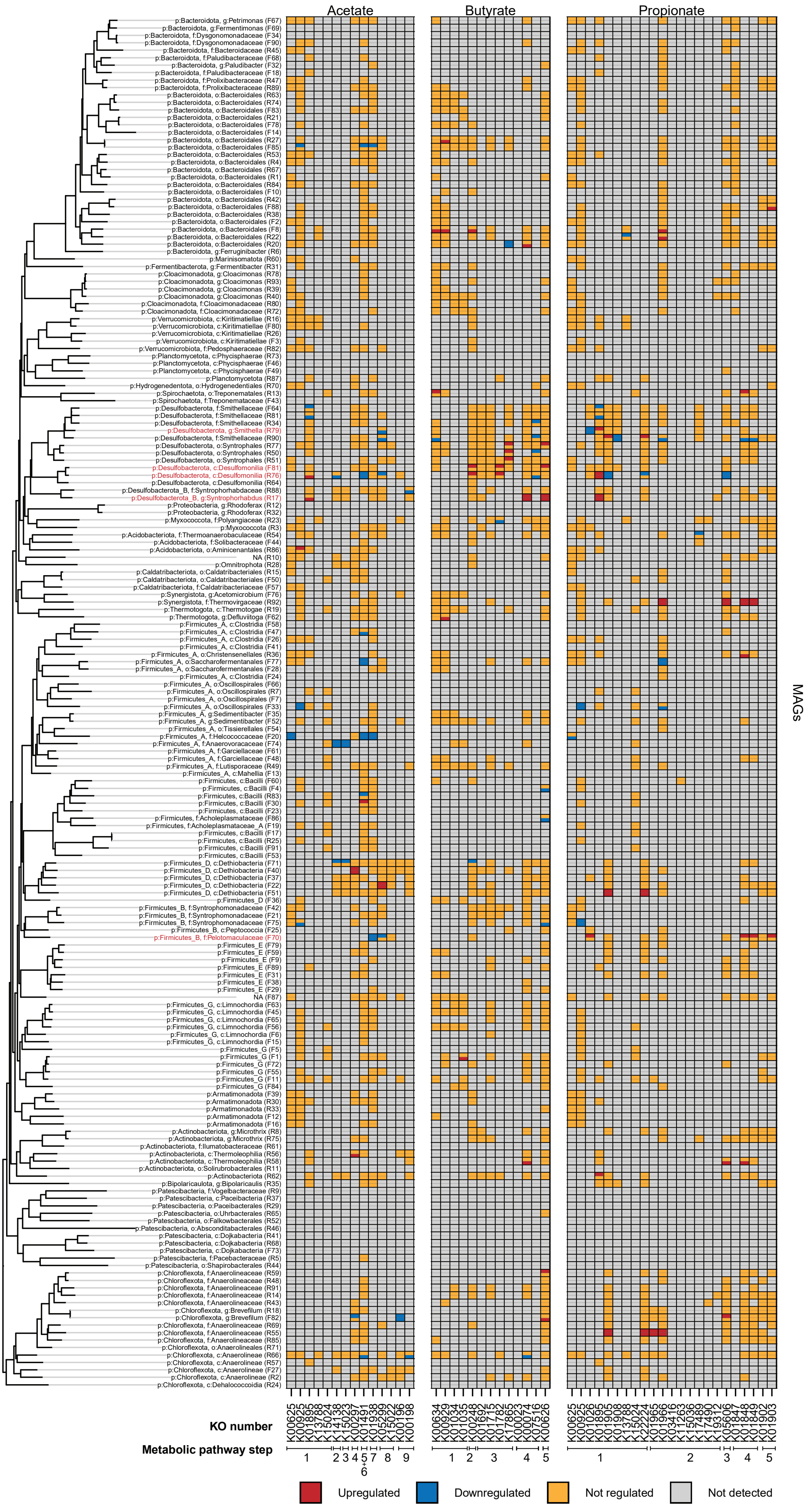

Acetate
Butyrate
Propionate
MAGs
KO number
Metabolic pathway step
Upregulated
Downregulated
Not regulated
Not detected

Figure S2. Transcriptional response of 184 bacterial MAGs after the SCFA stimulation. A phylogenetic tree of the MAGs is shown to the left. It is identical to the overall genome tree in Figure 2. The heatmap shows the transcriptional response of genes involved in the bioconversion of SCFAs in the MAGs. The target genes are described in Figure S1 and Data S2. The heatmap is colored according to fold change (FC) and the corresponding p-value (not corrected for multiple testing, to increase sensitivity) of CDSs for the given KO and are categorized as follows: Down-regulated: $FC \leq 0.5$ and $p\text{-value} < 0.05$; Up-regulated: $FC \geq 2$ and $p\text{-value} < 0.05$; Not regulated: $0.5 < FC < 2$ or $p > 0.05$; Not detected: no expression or not encoded. For each MAG, the corresponding SCFA stimuli and control conditions are shown in the upper- and lower-half of the tile, respectively. The potential syntrophs were highlighted by red font color of the taxonomic information, including the newly discovered and the ones belonging to the genera containing typical known syntrophic bacteria.

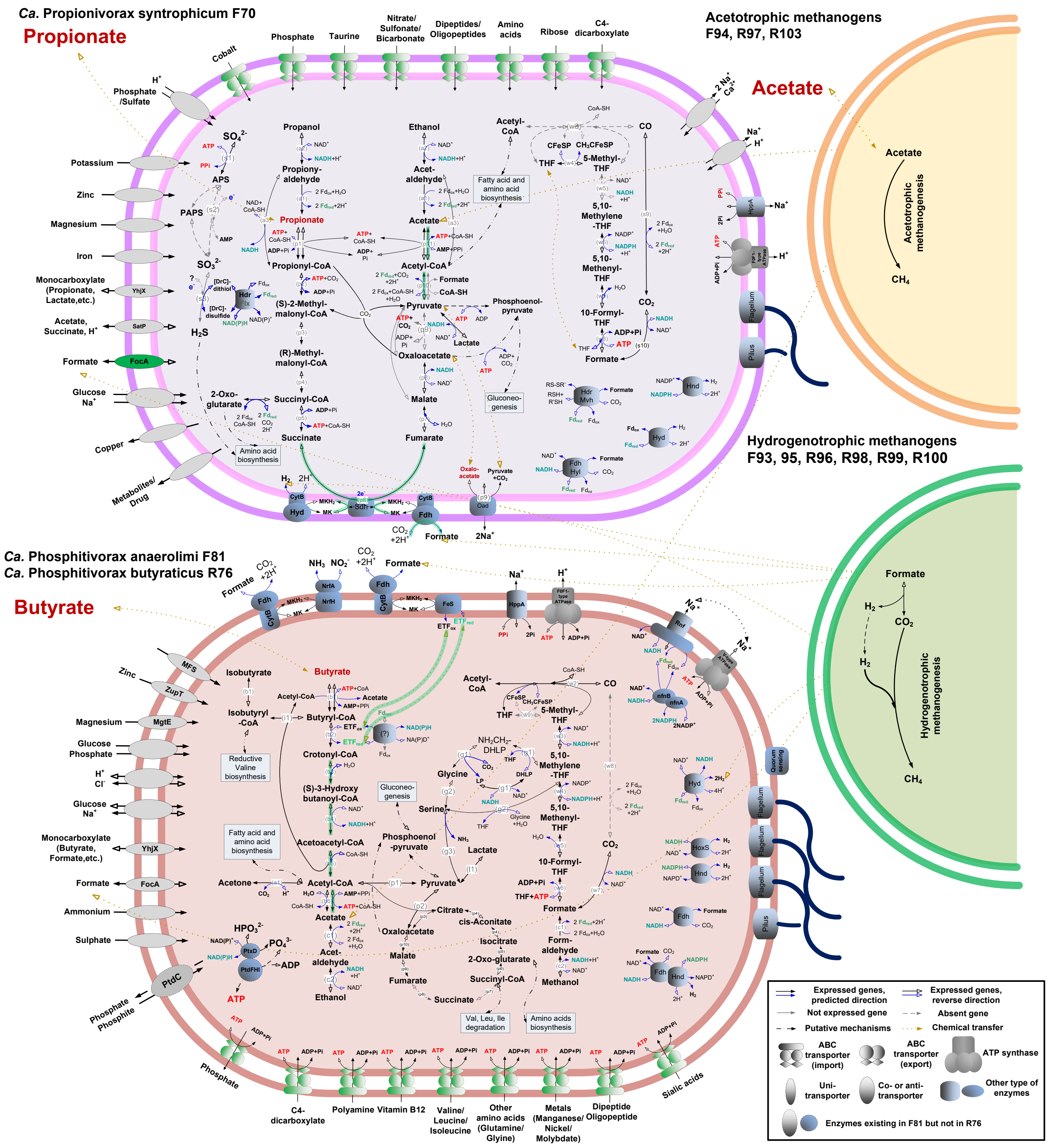

Ca. Propionivorax syntrophicum F70
Propionate
Acetotrophic methanogens
F94, R97, R103
Acetate
Acetotrophic methanogenesis
CH4
Hydrogenotrophic methanogens
F93, 95, R96, R98, R99, R100
Formate
H2
CO2
Hydrogenotrophic methanogenesis
Ca. Phosphitivorax anaerolimi F81
Ca. Phosphitivorax butyraticus R76
Butyrate
Phosphate
Taurine
Nitrate/ Sulfonate/ Bicarbonate
Dipeptides/ Oligopeptides
Amino acids
Ribose
C4-dicarboxylate
Cobalt
Phosphate /Sulfate
Potassium
Zinc
Magnesium
Iron
Monocarboxylate (Propionate, Lactate,etc.)
Acetate, Succinate, H+
Glucose
Copper
Metabolites/ Drug
Propanol
Propionyl-aldehyde
Propionyl-CoA
(S)-2-Methyl-malonyl-CoA
(R)-Methyl-malonyl-CoA
Succinyl-CoA
Succinate
2-Oxo-glutarate
Ethanol
Acet-aldehyde
Acetyl-CoA
Pyruvate
Phosphoenol-pyruvate
Lactate
Oxaloacetate
Malate
Fumarate
Gluconeo-genesis
Fatty acid and amino acid biosynthesis
Amino acid biosynthesis
CO
5-Methyl-THF
5,10-Methylene-THF
5,10-Methenyl-THF
10-Formyl-THF
Isobutyrate
Isobutyryl-CoA
Butyryl-CoA
Crotonyl-CoA
(S)-3-Hydroxy butanoyl-CoA
Acetoacetyl-CoA
Acetone
Reductive Valine biosynthesis
Glycine
Serine
Citrate
cis-Aconitate
Isocitrate
2-Oxo-glutarate
Succinyl-CoA
Form-aldehyde
Methanol
Val, Leu, Ile degradation
Amino acids biosynthesis
Sulphate
Ammonium
Phosphate Phosphite
Sialic acids
Polyamine
Vitamin B12
Valine/ Leucine/ Isoleucine
Other amino acids (Glutamine/ Glyine)
Metals (Manganese/ Nickel/ Molybdate)
Dipeptide Oligopeptide
Expressed genes, predicted direction
Expressed genes, reverse direction
Not expressed gene
Absent gene
Putative mechanisms
Chemical transfer
ABC transporter (import)
ABC transporter (export)
ATP synthase
Uni-transporter
Co- or anti-transporter
Other type of enzymes
Enzymes existing in F81 but not in R76

Figure S3. **Metabolic pathways in *Ca.* Propionivorax syntrophicum F70, *Ca.* Phosphitivorax anaerolimi F81 and *Ca.* Phosphitivorax butyraticus R76, and their interactions with methanogens.** The pathways are constructed based on the annotated genome sequences (Data S4). Orange and green cell cartoons symbolize MAGs of *Methanothrix* (F94, R97, R101, R103) consuming acetate and MAGs of *Methanoculleus* (F93, F95, R98, R99) and Methanoregulaceae (R100) utilizing H_2 and formate produced by the bacteria. The reactions and uni-transporters highlighted in light green indicate that expression of the related genes was upregulated after addition of the corresponding SCFA. Rnf = *Rhodobacter* nitrogen fixation complex; Nfn = NADH-dependent Fd_{red}:$NADP^+$ oxidoreductase; ATP = Adenosine triphosphate; CoA = Coenzyme A; THF = Tetrahydrofolate; NAD(P)H = Nicotinamide adenine dinucleotide (phosphate) hydrogen; Pi = Phosphate; PPi = Pyrophosphate; MFS = Major facilitator superfamily transporter

Supplementary Tables

Table S1. Characteristics of the two anaerobic digesters investigated. The two full-scale anaerobic digesters investigated are located at municipal WWTPs at Randers and Fredericia and have been operated for more than 5 years under stable conditions. The digester at Randers WWTP treats surplus activated sludge (BS) and primary sludge (PS), and the digester at Fredericia digests surplus activated sludge pretreated by thermal hydrolysis process (THP). They represent two types of mesophilic anaerobic digestion processes commonly used in Denmark. Median values during 2016 were used. PS: Primary Sludge; BS: Biological Sludge.

	**Randers**	**Fredericia**
Reactor type	CSTR	CSTR
Temperature	Mesophilic	Mesophilic
Sludge pretreatment	–	Thermal Hydrolysis Process (THP)
Feedstock	PS+BS	Hydrolyzed BS
Digesters	2	2
Temperature (°C)	39	38
Working Volume (m^3)	2400	2000
pH	7.4	7.8
Organic Loading Rate (kg-TS/(m^3·d))	3.2	3.4
Sludge Retention Time (d)	35	25
Volatile Solids Concentration (g/L)	22.8	25.2
TS reduction (%)	43	55
Gas production (m^3/(m^3·d))	0.51	1.17

Table S2. Generation of MAGs with MetaBAT2 and dRep. Several metagenomes were obtained for each digester, and these were assembled individually or as co-assemblies before generation of genome bins and MAGs. Two co-assembly settings were used: For FredCo1, RandCo1, and RandCo3 broken pairs were not used, and mapping was set as: length 0.7, similarity 0.95. For FredCo2, RandCo2, and RandCo4, broken pairs were used for assembly, and mapping was set as: length 1.0, similarity 0.95. * Raw bins produced by MetaBAT2 before quality control. ** Medium to high-quality MAGs (Completeness > 50%, contamination <25%) after dereplication with dRep.

Digester	Assembly ID	Samples used for assembly	Number of bins*	Total number of bins*	MAGs after dereplication**
Fredericia	FredS3	Fred-3	49	491	95
	FredS6	Fred-6	54		
	FredS9	Fred-9	56		
	FredS12	Fred-12	69		
	FredCo1	Fred-3, 6, 9, 12	135		
	FredCo2	Fred-3, 6, 9, 12	128		
Randers	RandS1	Rand-1	73	1007	103
	RandS4	Rand-4	55		
	RandS7	Rand-7	65		
	RandS10	Rand-10	47		
	RandCo1	Rand-1, 4, 7, 10	181		
	RandCo2	Rand-1, 4, 7, 10	186		
	RandS362	Rand-362	55		
	RandS363	Rand-363	58		
	RandS383	Rand-383	16		
	RandS384	Rand-384	23		
	RandCo3	Rand-362, 363, 383, 384	125		
	RandCo4	Rand-362, 363, 383, 384	123		

Table S3. Statistics of the metagenomes and DNA reads mapped to the MAGs. Trimmed reads from each metagenome were mapped to the 95 and 103 **dereplicated** MAGs from Fredericia or Randers respectively. In order to estimate the percentage of the microbial community represented by the MAGs, we calculated the percentage of the reads that were mapped to these MAGs.

Digester	Sample-ID	Total trimmed DNA Reads	Average length (bp)	Number of base pairs	DNA reads mapped to MAGs	Percent of DNA reads mapped to MAGs
Fredericia	Fred-3	17429764	227	3.96E+09	11380181	65%
	Fred-6	20045320	222	4.45E+09	11343248	57%
	Fred-9	23440250	216	5.07E+09	14504284	62%
	Fred-12	35858498	207	7.41E+09	20034092	56%
Randers	Rand-1	37121714	225	8.34E+09	16919325	46%
	Rand-4	23009028	222	5.11E+09	9811320	43%
	Rand-7	38047024	223	8.50E+09	15196143	40%
	Rand-10	21789328	223	4.86E+09	8474243	39%
	Rand-362	27571509	223	6.14E+09	10718531	39%
	Rand-363	38331609	209	7.99E+09	16410287	43%
	Rand-383	10128405	212	2.15E+09	3854556	38%
	Rand-384	12515654	211	2.64E+09	4638649	37%

Table S4. **Statistics of the metatranscriptomes and mRNA reads mapped to the CDSs predicted from the MAGs.** The rRNA reads were removed from the metatranscriptomes. The cDNA reads (translated from mRNA), after trimming and filtration of rRNA, are considered as mRNA and mapped to the CDSs predicted from 95 (Fredericia) and 103 (Randers) targeted MAGs. 1.7% and 3.9% of these reads were mapped to16S or 23S rRNA, indicating high rRNA removal efficiency during the RNA treatment process. These rRNA reads were filtered out, keeping only the mRNA reads for further use. Average values and standard deviation of the three biological replicates for each condition are shown in the lower part of the table.

**Sample ID**	**substrate**	**trimmed total RNA reads**		**Percent of rRNA reads**		**mRNA reads**		**Percent of mRNA reads mapped to CDSs**	
		Fredericia	Randers	Fredericia	Randers	Fredericia	Randers	Fredericia	Randers
Before 1	None	9875668	18437078	2.02	2.02	9676232	17803673	12.78	9.34
Before 2	None	12072577	18664545	1.40	5.79	11903873	17583489	12.31	9.66
Before 3	None	11608984	18503747	1.97	3.11	11380337	17928914	11.83	9.53
Acetate 1	Acetate	11824058	17086449	1.99	8.65	11589098	15607886	8.96	12.43
Acetate 2	Acetate	10614096	18285693	1.51	2.16	10453690	17891217	10.45	12.61
Acetate 3	Acetate	11424440	20031594	1.49	2.51	11253946	19529085	9.52	12.71
Propionate 1	Propionate	10136087	19025637	1.42	3.00	9992365	18455031	10.66	10.55
Propionate 2	Propionate	13286847	17703600	1.57	4.18	13078822	16964166	10.28	10.66
Propionate 3	Propionate	11506154	17807508	1.61	2.91	11320694	17288621	9.41	10.23
Butyrate 1	Butyrate	13745817	20013583	1.34	4.36	13561657	19140551	13.35	10.79
Butyrate 2	Butyrate	12616191	18532450	1.88	2.22	12379550	18120479	12.93	11.16
Butyrate 3	Butyrate	12199627	18803994	1.60	2.95	12004127	18248584	14.97	10.51
Control 1	Control	12367514	18079525	1.70	5.43	12157634	17098763	10.55	10.52
Control 2	Control	13232722	16273517	2.46	3.21	12907212	15750927	9.59	9.56
Control 3	Control	9960299	19512939	1.84	5.96	9776717	18350203	9.93	8.96
Average	None	11185743	18535123	1.80	3.64	10986814	17772025	12.31	9.51
Stdev.	None	1157995	116934	0.34	1.94	1164792	174874	0.48	0.16
Average	Acetate	11287531	18467912	1.66	4.44	11098911	17676063	9.64	12.58
Stdev.	Acetate	616490	1481004	0.28	3.65	583365	1969434	0.75	0.14
Average	Propionate	11643029	18178915	1.53	3.36	11463960	17569273	10.12	10.48
Stdev.	Propionate	1579833	735121	0.10	0.71	1548208	784056	0.64	0.22
Average	Butyrate	12853878	19116676	1.61	3.18	12648445	18503205	13.75	10.82
Stdev.	Butyrate	800030	788521	0.27	1.09	812837	555662	1.08	0.33
Average	Control	11853512	17955327	2.00	4.87	11613854	17066631	10.02	9.68
Stdev.	Control	1695682	1623278	0.40	1.46	1634556	1299936	0.49	0.79

Supplementary Datasets

Supplementary Data S1: Samples and sequencing data (separate file).

Supplementary Data S2: Functional gene families related to oxidation of propionate/butyrate/acetate (separate file).

Supplementary Data S3: Detailed information of 198 MAGs (separate file).

Supplementary Data S4: Expression data of the genes involved in the key metabolic pathways annotated for MAGs F70, F81 and R76 under different conditions (separate file).

Supplementary Data S5: Comparative genomic analysis between MAGs F70, F81, R76 with their closest relatives from GTDB (separate file).

Supplementary Data S6: Genome comparison with ANI between MAGs F70, F81, R76 with their closest relatives and the typical known SBOB and SPOB (separate file).

Supplementary References

Andersen KS, Kirkegaard RH, Karst SM, Albertsen M. (2018). ampvis2 : an R package to analyse and visualise 16S rRNA amplicon data. *bioRxiv* doi: http://dx.doi.org/10.1101/299537.

Bala JD, Lalung J, Ismail N. (2014). Palm oil mill effluent (POME) treatment "Microbial communities in an anaerobic digester": A Review. *Int J Sci Res Publ* **4**: 1–24.

Bar-Even A. (2016). Formate assimilation: The metabolic architecture of natural and synthetic pathways. *Biochemistry* **55**: 3851–3863.

Bar-Even A, Noor E, Flamholz A, Milo R. (2013). Design and analysis of metabolic pathways supporting formatotrophic growth for electricity-dependent cultivation of microbes. *Biochim Biophys Acta - Bioenerg* **1827**: 1039–1047.

de Bok FAM, Harmsen HJM, Plugge CM, de Vries MC, Akkermans ADL, de Vos WM, *et al.* (2005). The first true obligately syntrophic propionate-oxidizing bacterium, *Pelotomaculum schinkii* sp. nov., co-cultured with *Methanospirillum hungatei*, and emended description of the genus *Pelotomaculum*. *Int J Syst Evol Microbiol* **55**: 1697–1703.

Buckel W, Thauer RK. (2013). Energy conservation via electron bifurcating ferredoxin reduction and proton/Na^+ translocating ferredoxin oxidation. *Biochim Biophys Acta - Bioenerg* **1827**: 94–113.

Buckel W, Thauer RK. (2018). Flavin-based electron bifurcation, ferredoxin, flavodoxin, and anaerobic respiration with protons (Ech) or NAD^+ (Rnf) as electron acceptors: A historical review. *Front Microbiol* **9**: 401.

Campanaro S, Treu L, Kougias PG, Francisci D De, Valle G, De Francisci D, *et al.* (2016). Metagenomic analysis and functional characterization of the biogas microbiome using high throughput shotgun sequencing and a novel binning strategy. *Biotechnol Biofuels* **9**: 1–17.

Crable BR, Sieber JR, Mao X, Alvarez-Cohen L, Gunsalus R, Loo RRO, *et al.* (2016). Membrane complexes of *Syntrophomonas wolfei* involved in syntrophic butyrate degradation and hydrogen formation. *Front Microbiol* **7**: 1795.

Cracan V, Banerjee R. (2012). Novel B_{12}-dependent acyl-CoA mutases and their biotechnological potential. *Biochemistry* **51**: 6039–6046.

Djao ODN, Zhang X, Lucas S, Lapidus A, del Rio TG, Nolan M, *et al.* (2010). Complete genome sequence of *Syntrophothermus lipocalidus* type strain (TGB-C1^T). *Stand Genomic Sci* **3**: 267–275.

Eddy SR. (2011). Accelerated profile HMM searches. *PLoS Comput Biol* **7**: e1002195.

Edgar RC. (2018). Accuracy of taxonomy prediction for 16S rRNA and fungal ITS sequences. *PeerJ* **6**: e4652.

Edgar RC. (2010). Search and clustering orders of magnitude faster than BLAST. *Bioinformatics* **26**: 2460–2461.

Edgar RC. (2016). UNOISE2 : improved error-correction for Illumina 16S and ITS amplicon sequencing. *bioRxiv* doi: http://dx.doi.org/10.1101/081257.

Figueroa IA, Barnum TP, Somasekhar PY, Carlström CI, Engelbrektson AL, Coates JD. (2018). Metagenomics-guided analysis of microbial chemolithoautotrophic phosphite oxidation yields evidence of a

seventh natural CO_2 fixation pathway. *Proc Natl Acad Sci* **115**: E92–E101.

Grein F, Ramos AR, Venceslau SS, Pereira IAC. (2013). Unifying concepts in anaerobic respiration: Insights from dissimilatory sulfur metabolism. *Biochim Biophys Acta - Bioenerg* **1827**: 145–160.

Hedderich R, Hamann N, Bennati M. (2005). Heterodisulfide reductase from methanogenic archaea: A new catalytic role for iron-sulfur cluster. *Biol Chem* **386**: 961–970.

Hidalgo-ahumada CAP, Nobu MK, Narihiro T, Tamaki H, Liu W, Kamagata Y, *et al.* (2018). Novel energy conservation strategies and behaviour of Pelotomaculum schinkii driving syntrophic propionate catabolism. **20**: 4503–4511.

Hyatt D, Chen G-L, Locascio PF, Land ML, Larimer FW, Hauser LJ. (2010). Prodigal: prokaryotic gene recognition and translation initiation site identification. *BMC Bioinformatics* **11**: 119.

Imachi H, Sekiguchi Y, Kamagata Y, Hanada S, Ohashi A, Harada H. (2002). *Pelotomaculum thermopropionicum* gen . nov ., sp . nov ., an anaerobic , thermophilic , syntrophic propionate-oxidizing bacterium. *Int J Syst Evol Microbiol* **52**: 1729–1735.

Jackson BE, Bhupathiraju VK, Tanner RS, Woese CR, McInerney MJ. (1999). *Syntrophus aciditrophicus* sp. nov., a new anaerobic bacterium that degrades fatty acids and benzoate in syntrophic association with hydrogen-using microorganisms. *Arch Microbiol* **171**: 107–114.

Jain C, Rodriguez-r LM, Aluru S. (2018). High throughput ANI analysis of 90K prokaryotic genomes reveals clear species boundaries. *Nat Commun* **9**: 5114.

Juteau P, Côté V, Duckett MF, Beaudet R, Lépine F, Villemur R, *et al.* (2005). *Cryptanaerobacter phenolicus* gen. nov., sp. nov., an anaerobe that transforms phenol into benzoate via 4-hydroxybenzoate. *Int J Syst Evol Microbiol* **55**: 245–250.

Kirkegaard RH, Dueholm MS, McIlroy SJ, Nierychlo M, Karst SM, Albertsen M, *et al.* (2016). Genomic insights into members of the candidate phylum Hyd24-12 common in mesophilic anaerobic digesters. *ISME J* **10**: 1–13.

Kirkegaard RH, McIlroy SJ, Kristensen JM, Nierychlo M, Karst SM, Dueholm MS, *et al.* (2017). The impact of immigration on microbial community composition in full-scale anaerobic digesters. *Sci Rep* **7**: 9343.

Konstantinidis KT, Tiedje JM. (2005). Genomic insights that advance the species definition for prokaryotes. *Proc Natl Acad Sci U S A* **102**: 2567–2572.

Kosaka T, Kato S, Shimoyama T, Ishii S, Abe T, Watanabe K. (2008). The genome of *Pelotomaculum thermopropionicum* reveals niche-associated evolution in anaerobic microbiota. *Genome Res* **18**: 442–448.

Kouzuma A, Kato S, Watanabe K. (2015). Microbial interspecies interactions: recent findings in syntrophic consortia. *Front Microbiol* **6**: 477.

Letunic I, Bork P. (2016). Interactive tree of life (iTOL) v3: an online tool for the display and annotation of phylogenetic and other trees. *Nucleic Acids Res* **44**: W242–W245.

Ludwig W, Strunk O, Westram R, Richter L, Meier H, Yadhukumar A, *et al.* (2004). ARB: a software environment for sequence data. *Nucleic Acids Res* **32**: 1363–1371.

Matihiest C, Schinkv B, Matthies C, Schink B. (1992). Reciprocal isomerization of butyrate and isobutyrate

by the strictly anaerobic bacterium strain WoG13 and methanogenic isobutyrate degradation by a defined triculture. *Appl Environ Microbiol* **58**: 1435–1439.

McIlroy SJ, Kirkegaard RH, Dueholm MS, Fernando E, Karst SM, Albertsen M, *et al.* (2017). Culture-independent analyses reveal novel Anaerolineaceae as abundant primary fermenters in anaerobic digesters treating waste activated sudge. *Front Microbiol* **8**: 1134.

McInerney MJ, Bryant MP, Hespell RB, Costerton JW. (1981). *Syntrophomonas wolfei* gen. nov. sp. nov., an anaerobic, syntrophic, fatty acid-oxidizing bacterium. *Appl Environ Microbiol* **41**: 1029–1039.

McInerney MJ, Rohlin L, Mouttaki H, Kim U, Krupp RS, Rios-hernandez L, *et al.* (2007). The genome of *Syntrophus aciditrophicus*: Life at the thermodynamic limit of microbial growth. *Proc Natl Acad Sci U S A* **104**: 7600–7605.

Müller B, Sun L, Schnürer A. (2013). First insights into the syntrophic acetate-oxidizing bacteria--a genetic study. *Microbiologyopen* **2**: 35–53.

Müller N, Worm P, Schink B, Stams AJM, Plugge CM. (2010). Syntrophic butyrate and propionate oxidation processes: From genomes to reaction mechanisms. *Environ Microbiol Rep* **2**: 489–499.

Narihiro T, K. Nobu M, Tamaki H, Kamagata Y, Sekiguchi Y, Liu W-T. (2016). Comparative genomics of syntrophic branched-chain fatty acid degrading bacteria. *Microbes Environ* **31**: 288–292.

Nobu MK, Narihiro T, Hideyuki T, Qiu YL, Sekiguchi Y, Woyke T, *et al.* (2015a). The genome of *Syntrophorhabdus aromaticivorans* strain UI provides new insights for syntrophic aromatic compound metabolism and electron flow. *Environ Microbiol* **17**: 4861–4872.

Nobu MK, Narihiro T, Rinke C, Kamagata Y, Tringe SG, Woyke T, *et al.* (2015b). Microbial dark matter ecogenomics reveals complex synergistic networks in a methanogenic bioreactor. *ISME J* **9**: 1710–1722.

Nölling J, Breton G, Omelchenko M V, Kira S, Zeng Q, Gibson R, *et al.* (2001). Genome sequence and comparative analysis of the solvent-producing bacterium *Clostridium acetobutylicum*. *J Bacteriol* **183**: 4823–4838.

Oude Elferink SJWH, Lens PNL, Dijkema C, Stams AJMM. (1996). Isomerization of butyrate to isobutyrate by *Desulforhabdus amnigenus*. *FEMS Microbiol Lett* **142**: 237–241.

Parks DH, Chuvochina M, Waite DW, Rinke C, Skarshewski A, Chaumeil P-A, *et al.* (2018). A standardized bacterial taxonomy based on genome phylogeny substantially revises the tree of life. *Nat Biotechnol* **36**: 996–1004.

Parks DH, Rinke C, Chuvochina M, Chaumeil P-AA, Woodcroft BJ, Evans PN, *et al.* (2017). Recovery of nearly 8,000 metagenome-assembled genomes substantially expands the tree of life. *Nat Microbiol* **2**: 1533–1542.

Quast C, Pruesse E, Yilmaz P, Gerken J, Schweer T, Glo FO, *et al.* (2013). The SILVA ribosomal RNA gene database project: improved data processing and web-based tools. *Nucleic Acids Res* **41**: 590–596.

R Core Team. (2017). R: a language and environment for statistical computing. R Foundation for Statistical Computing, Vienna: Vienna, Austria.

Ragsdale SW, Pierce E. (2009). Acetogenesis and the wood-ljungdahl pathway of CO_2 fixation. *Biochim Biophys Acta* **1784**: 1873–1898.

Ramos AR, Grein F, Oliveira GP, Venceslau SS, Keller KL, Wall JD, *et al.* (2015). The FlxABCD-HdrABC proteins correspond to a novel NADH dehydrogenase/heterodisulfide reductase widespread in anaerobic bacteria and involved in ethanol metabolism in *Desulfovibrio vulgaris* Hildenborough. *Environ Microbiol* **17**: 2288–2305.

Richter M, Rosselló-Móra R, Oliver Glöckner F, Peplies J. (2015). JSpeciesWS: A web server for prokaryotic species circumscription based on pairwise genome comparison. *Bioinformatics* **32**: 929–931.

Sedano-Núñez VT, Boeren S, Stams AJM, Plugge CM. (2018). Comparative proteome analysis of propionate degradation by *Syntrophobacter fumaroxidans* in pure culture and in coculture with methanogens. *Environ Microbiol* **20**: 1842–1856.

Seemann T. (2014). Prokka: Rapid prokaryotic genome annotation. *Bioinformatics* **30**: 2068–2069.

Sekiguchi Y, Kamagata Y, Nakamura K, Ohashi A, Harada H. (2000). *Syntrophothermus lipocalidus* gen. nov., sp. nov., a novel thermophilic, syntrophic, fatty-acid-oxidizing anaerobe which utilizes isobutyrate. *Int J Syst Evol Microbiol* **50**: 771–779.

Sieber JR, Crable BR, Sheik CS, Hurst GB, Rohlin L, Gunsalus RP, *et al.* (2015). Proteomic analysis reveals metabolic and regulatory systems involved in the syntrophic and axenic lifestyle of *Syntrophomonas wolfei*. *Front Microbiol* **6**: 115.

Sieber JR, Sims DR, Han C, Kim E, Lykidis A, Lapidus AL, *et al.* (2010). The genome of *Syntrophomonas wolfei*: new insights into syntrophic metabolism and biohydrogen production. *Environ Microbiol* **12**: 2289–2301.

Stams AJM, Plugge CM. (2009). Electron transfer in syntrophic communities of anaerobic bacteria and archaea. *Nat Rev Microbiol* **7**: 568–577.

Visser M, Worm P, Muyzer G, Pereira IAC, Schaap PJ, Plugge CM, *et al.* (2013). Genome analysis of *Desulfotomaculum kuznetsovii* strain 17^T reveals a physiological similarity with *Pelotomaculum thermopropionicum* strain SI^T. *Stand Genomic Sci* **8**: 69–87.

Wang S, Huang H, Kahnt J, Thauer RK. (2013). *Clostridium acidurici* electron-bifurcating formate dehydrogenase. *Appl Environ Microbiol* **79**: 6176–6179.

Yilmaz P, Parfrey LW, Yarza P, Gerken J, Pruesse E, Quast C, *et al.* (2014). The SILVA and "All-species Living Tree Project (LTP)" taxonomic frameworks. *Nucleic Acids Res* **42**: D643–D648.
